# PLK1 or WEE1 inhibition targets homologous recombination repair proficiency in *BRCA1*/*2* wild-type high-grade serous ovarian cancer

**DOI:** 10.1038/s41419-025-08324-2

**Published:** 2025-12-07

**Authors:** Qian Xi, Akiko Kunita, Miho Ogawa, Masanori Kawakami, Mirei Ka, Saeko Nagai, Anh Quynh Duong, Ayumi Taguchi, Kousuke Watanabe, Tomohiko Fukuda, Kenbun Sone, Aya Shinozaki-Ushiku, Tetsuo Ushiku, Yasushi Hirota, Hidenori Kage, Kazuhiro Katayama, Katsutoshi Oda

**Affiliations:** 1 Division of Integrative Genomics, Graduate School of Medicine, The University of Tokyo, Tokyo, Japan; 2 Department of Gynecological Oncology, Sun Yat-sen University Cancer Center, Guangzhou, China; 3 Next-Generation Precision Medicine Development Laboratory, Graduate School of Medicine, The University of Tokyo, Tokyo, Japan; 4 Department of Pathology, Graduate School of Medicine, The University of Tokyo, Tokyo, Japan; 5 Department of Respiratory Medicine, Graduate School of Medicine, The University of Tokyo, Tokyo, Japan; 6 Department of Obstetrics and Gynecology, Graduate School of Medicine, The University of Tokyo, Tokyo, Japan; 7 Department of Obstetrics and Gynecology, Faculty of Medicine, University of Yamanashi, Yamanashi, Japan; 8 Laboratory of Molecular Targeted Therapeutics, School of Pharmacy, Nihon University, Chiba, Japan

**Keywords:** Targeted therapies, Predictive markers, Homologous recombination, Preclinical research, Ovarian cancer

## Abstract

High-grade serous ovarian cancer (HGSOC) is a poor prognostic disease, especially in *BRCA1*/*2* wild-type (BRCA-WT) patients with homologous recombination (HR) proficiency. These patients often show limited response to both platinum-based chemotherapy and PARP inhibitors. HR and non-homologous end joining (NHEJ) are the two major DNA double-strand break (DSB) repair pathways. HR is a precise repair mechanism for DSBs but is limited to S and G2 phases. In contrast, NHEJ functions more broadly throughout the cell cycle, including G1. We investigated whether inhibiting the G2/M checkpoint kinases PLK1 or WEE1 individually could disrupt mitotic control and expose therapeutic vulnerabilities in BRCA-WT/HR-proficient HGSOC cells. We evaluated cell cycle–targeted strategies to overcome HR-proficient chemoresistance using either volasertib (a selective PLK1 inhibitor) or adavosertib (a potent WEE1 inhibitor) in BRCA-WT/HR-proficient and BRCA-mutant/HR-deficient HGSOC models. Both agents induced DNA damage, impaired HR repair (reduced RAD51 foci), and triggered mitotic catastrophe—a form of cell death caused by defective mitosis and unresolved DNA damage—in BRCA-WT cells. Volasertib caused polyploidy and abnormal spindle formation, indicating mitotic slippage and cytokinesis failure, whereas adavosertib abrogated the G2/M checkpoint, forcing premature mitotic entry. In contrast, BRCA-mutant cells were resistant to either volasertib or adavosertib, consistent with sustained and functional NHEJ activity. This resistance was restored by the pharmacological or genetic inhibition of DNA-PKcs (DNA-dependent protein kinase, catalytic subunit), a prominent component of NHEJ. Functional and xenograft models confirmed selective vulnerability of BRCA-WT tumors to either PLK1 or WEE1 inhibition. Our work highlights a mechanistic framework linking cell cycle checkpoint inhibition to DNA repair pathway selectivity, providing a rationale for targeting mitotic regulators in HR-proficient ovarian cancer—a subgroup with high clinical unmet need.

## Introduction

Ovarian cancer, the third most prevalent gynecological malignancy worldwide, is characterized by high lethality and poor prognosis, with a high prevalence of advanced-stage cancer at initial diagnosis [1]. High-grade serous ovarian cancer (HGSOC) is the most common histological subtype of ovarian cancer, accounting for the majority of ovarian cancer-related deaths. HGSOC is frequently associated with *BRCA1/2* mutations that disrupt homologous recombination (HR), a key DNA repair pathway. These mutations sensitize tumors to DNA-damaging agents including platinum-based chemotherapy and poly (ADP-ribose) polymerase (PARP) inhibitors [2]. The SOLO-1 trial showed that olaparib maintenance significantly improved progression-free survival, with a 7-year overall survival of 67% in newly diagnosed *BRCA1*/*2*-mutated (BRCA-Mut) ovarian carcinomas [3, 4]. Approximately 50% of advanced HGSOC cases are HR-deficient (HRD) based on MyChoice CDx testing [5–7], mainly due to *BRCA1*/*2* mutations (approximately 25–30%) or epigenetic alterations in *BRCA1* or *RAD51C* (10–15%) [8, 9]. HRD is also observed in tumors with unknown genetic or epigenetic alterations.

Conversely, the remaining ~50% of HGSOC cases are HR-proficient (HRP) and exhibit poor response to PARP inhibitors. Additionally, HRD status can be unstable and may revert to HRP through *BRCA1/2* reversion mutations or gene demethylation, contributing to acquired resistance [10, 11]. Although PARP inhibitor–based maintenance therapy has improved the outcomes of BRCA-Mut HGSOC, the prognosis of *BRCA1*/*2* wild-type (BRCA-WT) HGSOC, particularly in HRP cases, remains poor [12, 13]. In contrast to extensive research on HRD biology, effective therapies for BRCA-WT and HRP HGSOC remain limited, emphasizing the need for novel biomarkers and treatment approaches [14].

Polo-like kinase 1 (PLK1) is a key serine/threonine protein kinase found in various cancers, including ovarian cancer [15, 16]. PLK1 is essential for centrosome maturation, spindle assembly, chromosome segregation, and cytokinesis (proper mitotic progression) [17, 18]. PLK1 also regulates BRCA1/2 via phosphorylation and is vital for maintaining the G2/M checkpoint and genomic integrity [19, 20]. Moreover, PLK1 contributes to the regulation of the HR pathway by interacting with and phosphorylating key proteins such as RAD51 and PPIL2, thus promoting efficient DNA repair responses [21, 22]. Preclinical and early clinical studies have demonstrated the promising antitumor activity of PLK1 inhibitors in ovarian cancer [23–25]. However, in platinum-resistant or refractory ovarian cancer, clinical trials of PLK1 inhibitors such as volasertib have demonstrated only modest efficacy, with progression-free survival not superior to chemotherapy and frequent hematologic toxicities as the principal dose-limiting events [26, 27]. The absence of reliable biomarkers in these studies highlights the need for mechanistic insights and rational stratification to improve patient selection and therapeutic strategies.

The tyrosine kinase WEE1 is another critical kinase in ovarian cancer, and its overexpression is an independent marker of poor prognosis in HGSOC [28, 29]. WEE1 regulates G2/M phase transition during cell cycle regulation and DNA damage response, ensuring cell cycle fidelity. Furthermore, WEE1 is involved in HR repair regulation through CDK1-dependent phosphorylation of *BRCA2* [30]. CHK1 and CHK2 mediate cell cycle checkpoint activation, whereas RAD51 is central to HR repair [31, 32]. In HR-deficient cells, DNA double-strand break repair often shifts toward the error-prone non-homologous end joining (NHEJ) pathway, primarily mediated by DNA-PKcs [33–35]. A study showed that WEE1 inhibition sensitized *BRCA2* wild-type but not *BRCA2* mutant cancer cells to gemcitabine-based chemoradiation in pancreatic cancer cells [36]. WEE1 inhibitors such as adavosertib (AZD1775) have demonstrated clinical efficacy as monotherapy or in combination with carboplatin for platinum-resistant ovarian cancer [37–39]. Although *TP53* mutations have commonly been used as an inclusion criterion in clinical trials of WEE1 inhibitors, including HGSOC and uterine serous carcinoma, but it has not consistently predicted therapeutic benefit [38, 40]. Our previous work further demonstrated that *TP53* mutations alone may not be a reliable marker of sensitivity to WEE1 inhibition and that cyclin E1 overexpression sensitizes ovarian cancer cells to PLK1 and WEE1 inhibition, identifying its role as a predictive biomarker [41].

In this study, we focused on BRCA-WT and HRP HGSOC cells with low cyclin E1 expression, based on the hypothesis that PLK1 and WEE1 inhibition may provide a therapeutic window even in the absence of cyclin E1 overexpression—given the frequent *TP53* mutations and cell cycle dysregulation characteristic of HGSOC. Accordingly, we investigated the effects of PLK1 and WEE1 inhibition in BRCA-WT and BRCA-Mut HGSOC models, focusing on DNA repair dependency, cell cycle regulation, and differential drug sensitivity, to identify therapeutic strategies for BRCA-WT and HRP ovarian cancers.

## Results

### BRCA-WT HGSOC cells exhibit high sensitivity to PLK1 and WEE1 inhibition

We assessed *PLK1* and *WEE1* mRNA expression in ovarian cancer, predominantly HGSOC, and found that both genes were significantly overexpressed (*n* = 426) compared with normal ovarian tissues (*n* = 88) in the TCGA and GTEx datasets (Supplementary Fig. S1A), and were positively correlated with each other and with *BRCA1*/*2* expression (Supplementary Fig. S1B–D). These findings suggest a potential coordinated role for PLK1 and WEE1 in BRCA1/2-mediated DNA damage repair and ovarian tumorigenesis.

**Fig. 1:**
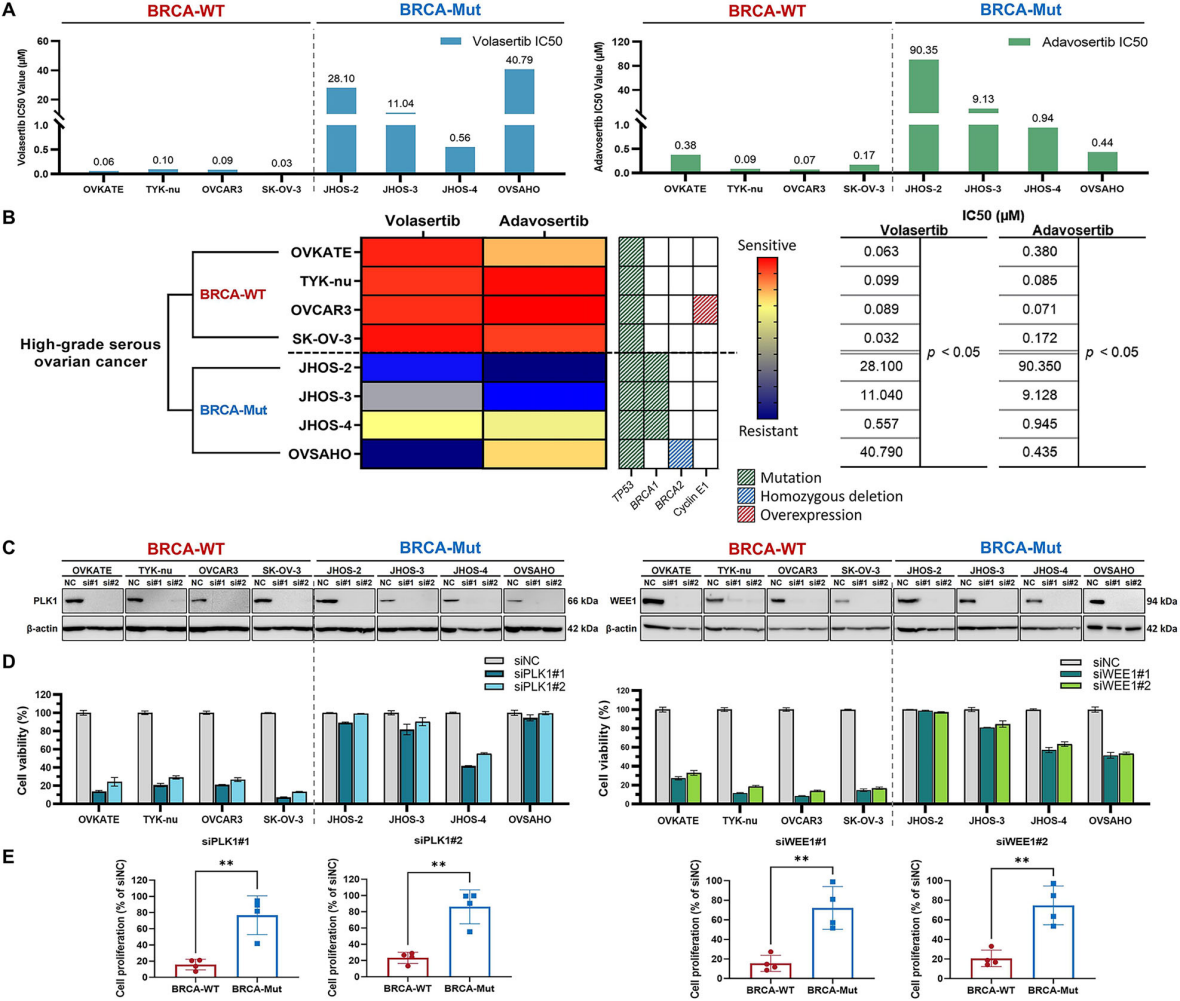
Impact of PLK1 and WEE1 silencing on cell proliferation and inhibitor sensitivity in BRCA-wild type ovarian cancer cells. **A**, **B** Sensitivity of HGSOC cell lines to the PLK1 inhibitor (volasertib) and WEE1 inhibitor (adavosertib), determined based on the half-maximal inhibitory concentration (72 h), and classified by *BRCA1/2* gene status. Mutation data were classified as pathogenic or likely pathogenic based on assessments of the COSMIC and ClinVar databases. **C**–**E** The efficiency of PLK1 and WEE1 knockdown in HGSOC cell lines was analyzed along with its subsequent impact on cell proliferation. HGSOC, high-grade serous ovarian cancer; BRCA-WT, *BRCA1/2* wild-type; BRCA-Mut, *BRCA1/2* mutant. IC_50_ values were calculated from the CCK-8 assay-based dose-response curves using GraphPad Prism. Data are presented as mean ± SD from three independent experiments. Statistical significance: **P < 0.01 using an unpaired two-tailed Student’s t-test.

Then, we tested sensitivity to PLK1 inhibitor volasertib and WEE1 inhibitor adavosertib across eight HGSOC cell lines, including four with *BRCA1/2* mutations (Supplementary Table S1). BRCA-WT cells showed higher sensitivity to both inhibitors than BRCA-Mut cells, with lower half-maximal inhibitory concentrations (IC50) of volasertib (median IC50 = 0.076 µM [95% CI: 0.032–0.099] vs. 19.57 µM [0.557–40.79]) and adavosertib (0.129 µM [0.071–0.380] vs. 5.04 µM [0.435–90.35]) against WT cells (Fig. 1A, B). Knocking down PLK1 or WEE1 by siRNA suppressed cell proliferation specifically in BRCA-WT HGSOC cells (Fig. 1C–E). These data suggest therapeutic benefit of targeting PLK1 and WEE1 in BRCA-WT HGSOC.

Given the involvement of PLK1 and WEE1 in HR repair, we assessed HR status using RAD51 foci formation in BRCA-WT cells (SK-OV-3 and TYK-nu) and BRCA-Mut cells (JHOS-2 with a *BRCA1* mutation and OVSAHO with a *BRCA2* homozygous deletion). RAD51 foci were present in BRCA-WT cells treated with cisplatin but absent in BRCA-Mut cells (Fig. S2), indicating HRP and HRD status, respectively. SK-OV-3 and TYK-nu cells, which lack cyclin E1 overexpression (Supplementary Table S1), were used as representative BRCA-WT cells.

### PLK1/WEE1 inhibition induces cell cycle dysregulation and apoptosis in BRCA-WT cells

**Fig. 2:**
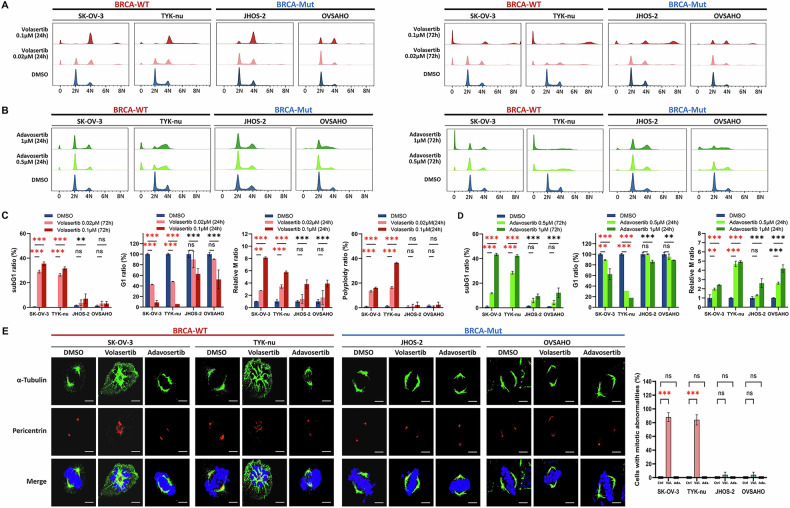
Cell cycle alterations induced by volasertib and adavosertib in BRCA wild-type ovarian cancer cells. **A**, **B** Representative flow cytometry plots showing the cell cycle profiles of BRCA wild-type (WT; SK-OV-3, TYK-nu) and mutant (Mut; JHOS-2, OVSAHO) ovarian cancer cells treated with control, low, or high concentrations of volasertib **A** or adavosertib **B** for 24 and 72 h. **C**, **D** Quantification of the cell cycle phase distribution following volasertib **C** or adavosertib **D** treatment. **E** Immunofluorescent staining of microtubules and centrosomes after treatment with volasertib or adavosertib. Cells were stained with α-tubulin (green), pericentrin (red), and DAPI (blue). Scale bar, 5 μm. Data are presented as mean ± SD from three independent experiments. Statistical significance: **P < 0.01; ***P < 0.001; ns, not significant using a one-way ANOVA.

Volasertib significantly increased the proportion of sub-G1 cells at 24 h, with a dose-dependent rise at 72 h, specifically in BRCA-WT cells (Fig. 2A, C), while adavosertib had a similar effect (Fig. 2B, D). Both drugs markedly reduced the G1 population and induced accumulation of M-phase cells, particularly in BRCA-WT cells (Fig. 2C, D). Considering the exclusive presence of a sub-G1 population in BRCA-WT cells, overriding the G1 checkpoint likely contributes to enhanced treatment sensitivity. Co-staining with cyclin A2 and propidium iodide confirmed accumulation of M-phase cells (Supplementary Fig. S3). Volasertib also induced polyploidy (>4 N DNA content) in BRCA-WT cells (Fig. 2C). Immunofluorescence revealed abnormal spindle structures, disrupted α-tubulin organization, and incomplete pericentrin separation in volasertib-treated BRCA-WT cells, but not after adavosertib treatment (Fig. 2E), indicating mitotic defects.

**Fig. 3:**
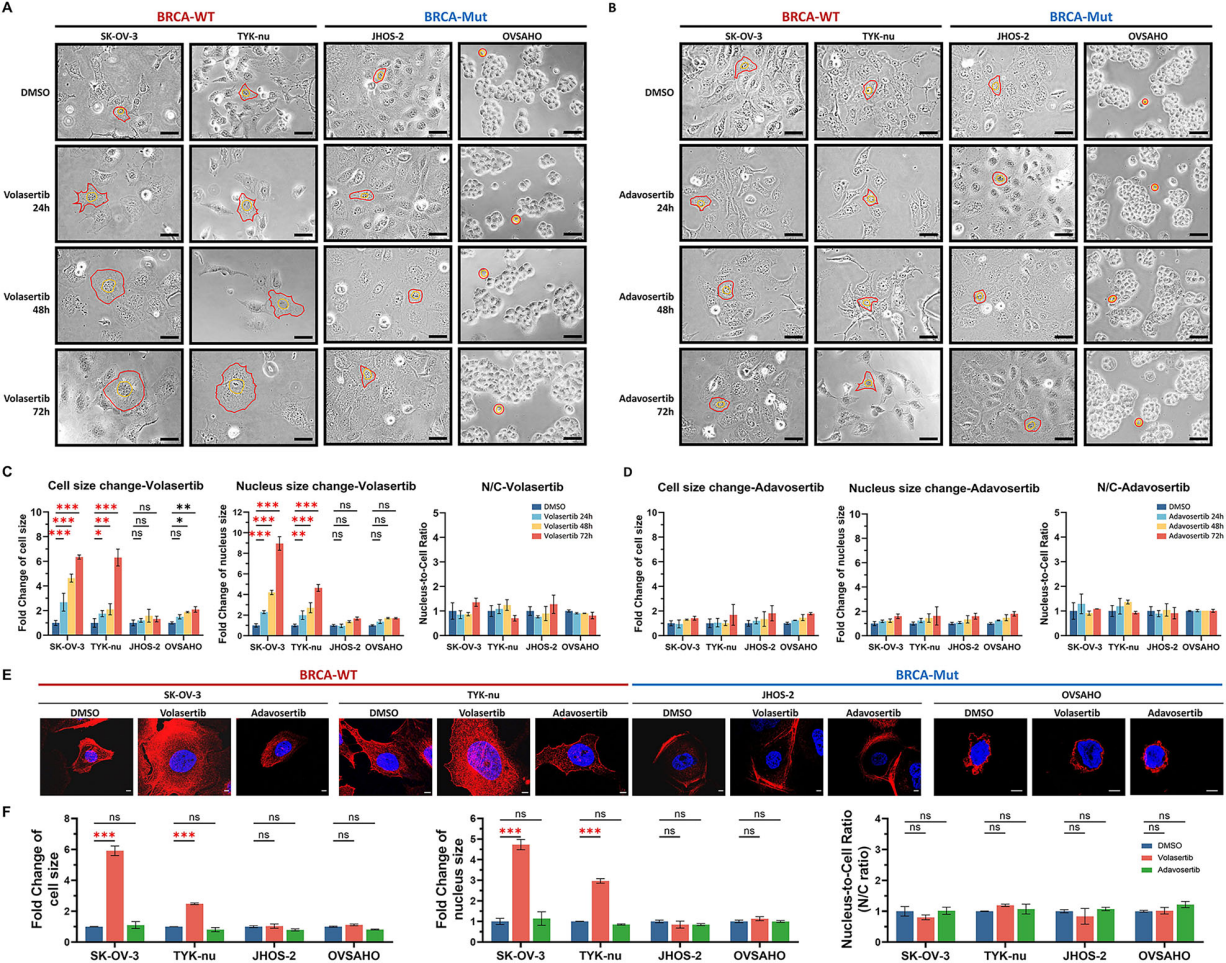
Morphological and apoptotic responses to PLK1 and WEE1 inhibition in BRCA wild-type ovarian cancer cells. **A**, **B** Representative images showing the morphology of BRCA-WT (SK-OV-3, TYK-nu) and BRCA-Mut (JHOS-2, OVSAHO) cells after treatment with volasertib and adavosertib. Scale bar = 50 µm. **C**, **D** Quantification of the nuclear area, overall cell size, and nuclear-to-cytoplasmic (N/C) ratio. **E**, **F** Representative fluorescence images of cells stained with phalloidin after 24-h treatment with volasertib or adavosertib, and corresponding quantification of cell area to assess cytoskeletal changes. Scale bar = 5 μm. Data are presented as mean ± SD from three independent experiments. Statistical significance: *P < 0.05, **P < 0.01, ***P < 0.001; ns, not significant using a two-way ANOVA.

Considering the link between cell cycle arrest and cellular morphology, we examined changes in cell and nuclear size following treatment. Flow cytometry forward scatter (FSC) plots showed a rightward shift, indicating an increase in cell size, particularly in BRCA-WT populations after volasertib treatment, whereas BRCA-Mut cells exhibited only a modest change (Supplementary Fig. S4). Side scatter (SSC) values also increased in BRCA-WT cells, indicating increased cytoplasmic complexity, likely related to microtubule polymerization during prolonged mitotic arrest. Image-based analysis at the single-cell level further confirmed proportional enlargement of cytoplasm and nucleus, consistent with cytokinesis failure caused by PLK1 inhibition (Fig. 3A–F).

### PLK1/WEE1 disruption leads to HR repair suppression in BRCA-WT cells and NHEJ-driven resistance in BRCA mutants

**Fig. 4:**
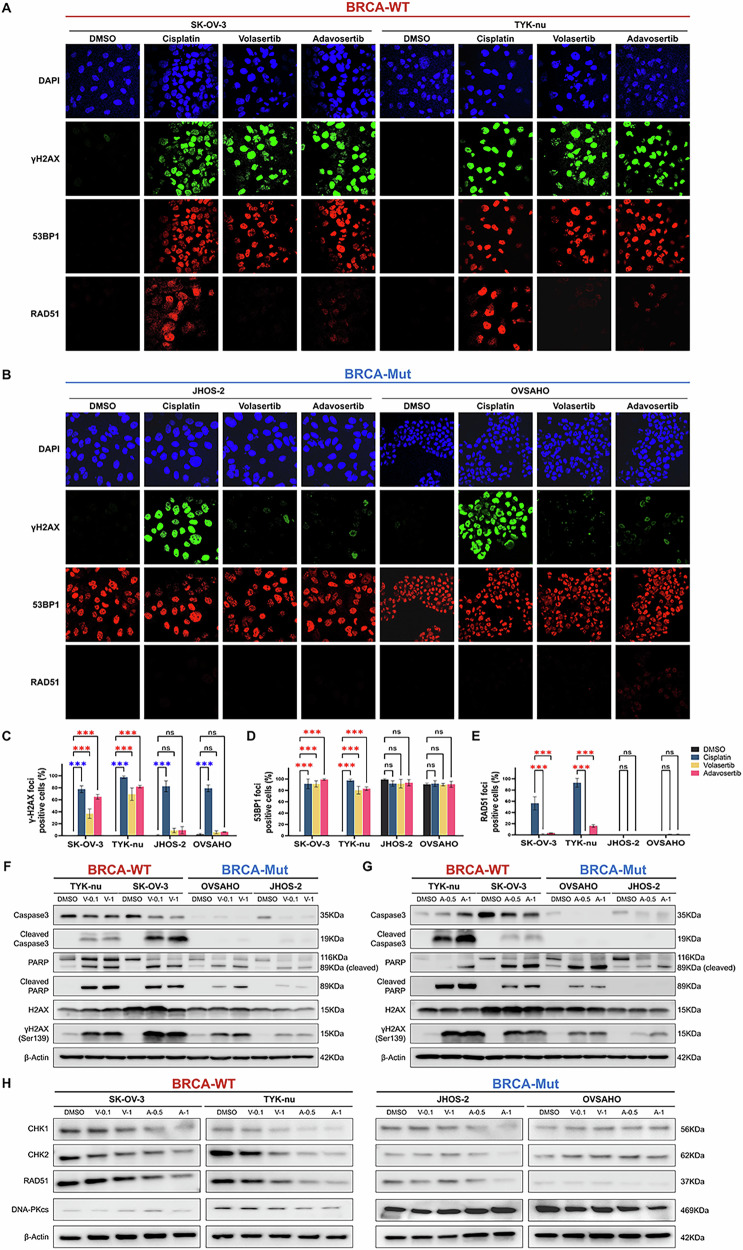
Analysis of DNA damage response pathways in BRCA wild-type and mutant ovarian cancer cells. **A**, **B** Immunofluorescence analysis of BRCA wild-type (WT) and mutant (Mut) cells treated with cisplatin, volasertib, or adavosertib for 24 h. Cells were stained with antibodies against γH2AX (a marker of DNA double-strand breaks), 53BP1 (a marker of non-homologous end joining, NHEJ), and RAD51 (a marker of homologous recombination, HR). **C–E** Quantification of γH2AX, 53BP1, and RAD51 foci. **F**, **G** Expression of apoptosis-related and DNA damage response proteins in BRCA-WT and -Mut cell lines following treatment with volasertib **F** or adavosertib **G**. **H** Immunoblot analysis of DNA damage response proteins, including CHK1, CHK2, RAD51, and DNA-PKcs, in BRCA-WT and BRCA-Mut cells after treatment with adavosertib for 24 h. β-actin was used as a loading control. V-0.1, volasertib 0.1 µM; V-1, volasertib 1 µM; A-0.5, adavosertib 0.5 µM; A-1, adavosertib 1 μM. Data are presented as mean ± SD from three independent experiments. Statistical significance: ***P < 0.001; ns, not significant using a two-way ANOVA.

Given the distinct apoptosis and cell cycle defects in BRCA-WT cells after PLK1 or WEE1 inhibition, we investigated whether they differentially utilized DNA repair pathways. Cisplatin, a well-established inducer of double-strand breaks (DSBs) and HR repair [42], was used as reference. PLK1 or WEE1 inhibition markedly increased γH2AX (Ser139) in BRCA-WT cells (*P* < 0.001) (Fig. 4A, C), but not in BRCA-Mut cells (Fig. 4B). To investigate the pathways involved in repairing these DSBs, we examined 53BP1, a marker of the NHEJ pathway [43]. In BRCA-WT cells, 53BP1 foci increased significantly (*P* < 0.001) following treatment with the inhibitors, indicating compensatory activation of NHEJ in response to HR suppression (Fig. 4A, D). However, BRCA-Mut cells showed constitutive 53BP1 foci unaffected by cisplatin or the PLK1/WEE1 inhibitors (Fig. 4B, D). Immunofluorescence assays revealed the absence of RAD51 foci formation, a key HR marker [44], in BRCA-Mut following cisplatin treatment, confirming their inherent HRD (Fig. 4B). In contrast, BRCA-WT cells formed RAD51 foci after cisplatin, confirming HR proficiency (Fig. 4A). Notably, volasertib or adavosertib significantly reduced (*P* < 0.001) HR repair activity in BRCA-WT cells, as evidenced by the loss of RAD51 foci (Fig. 4A, E). Furthermore, when cells were pretreated with cisplatin to induce DNA damage, subsequent exposure to the inhibitor markedly reduced RAD51 foci, confirming the suppression of HR repair by PLK1/WEE1 inhibition (Supplementary Fig. S5).

Apoptosis markers (cleaved PARP, caspase-3) increased in BRCA-WT cells following volasertib or adavosertib treatment, with weaker effects in BRCA-Mut cells (Fig. 4F, G), consistent with the dose-dependent increase in the sub-G1 fraction. γH2AX levels also increased markedly in BRCA-WT cells but remained low in BRCA-Mut cells, reflecting differential DSB accumulation (Fig. 4C, F,G). Analysis of DNA repair proteins showed that CHK1, CHK2, and RAD51, highly expressed at baseline in BRCA-WT cells, were markedly reduced after treatment, especially with adavosertib, whereas BRCA-Mut cells were largely unchanged (Fig. 4H). This downregulation, involving CHK1/CHK2 and RAD51, indicates collapse of the HR repair system and checkpoint signaling in BRCA-WT cells. In contrast, DNA-PKcs was low and unaltered in BRCA-WT cells but consistently high in BRCA-Mut cells, suggesting constitutive NHEJ activity. Overall, BRCA-WT cells showed HR suppression with limited NHEJ, while BRCA-Mut cells relied on constitutive NHEJ.

### BRCA-Mut cells exhibit a dependency on the NHEJ repair pathway

**Fig. 5:**
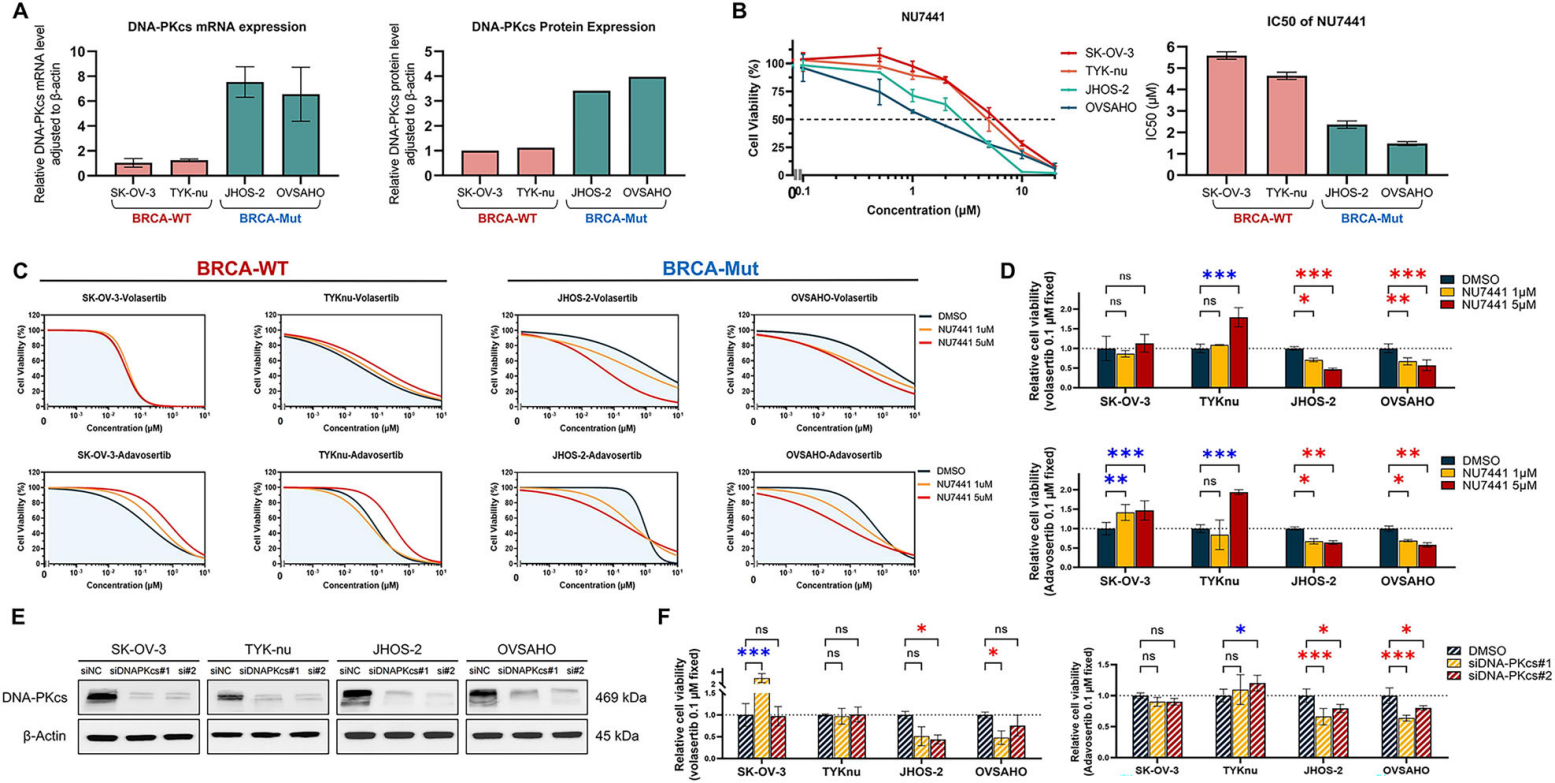
Non-homologous end joining (NHEJ) pathway inhibition and its impact on drug sensitivity in BRCA wild-type and mutant ovarian cancer cells. **A** DNA-PKcs mRNA and protein expression levels in BRCA-WT (SK-OV-3 and TYK-nu) and -Mut (JHOS-2 and OVSAHO) ovarian cancer cell lines. **B** Cell proliferation curves after treatment with the NHEJ inhibitor NU7441 for 72 h, along with the corresponding half-maximal inhibitory concentration (IC_50_) values shown in the bar graphs. **C**, **D** Sensitivity of cells to volasertib and adavosertib following 1-h pre-treatment with NU7441 at low (1 µM) and high (5 µM) concentrations, followed by volasertib or adavosertib treatment. **E**, **F** siRNA-mediated knockdown of DNA-PKcs and its effect on cell sensitivity to volasertib and adavosertib following 48-h transfection. Data are presented as mean ± SD from three independent experiments. Statistical significance: *P < 0.05, **P < 0.01, and ***P < 0.001; ns, not significant using a two-way ANOVA. Red asterisks indicate a significant decrease in cell viability compared to the control, suggesting increased sensitivity; blue asterisks indicate a significant increase, suggesting acquired resistance.

Considering the distinct DNA repair responses, we hypothesized that BRCA-Mut cells rely on the NHEJ pathway, particularly DNA-PKcs activity. The DNA-PKcs expression was higher in BRCA-Mut cells than in BRCA-WT cells at mRNA and protein levels (Fig. 5A). BRCA-Mut cells showed stronger sensitivity to DNA-PKcs inhibitor NU7441 than BRCA-WT cells (Fig. 5B). NU7441 pre-treatment significantly increased BRCA-Mut cells’ sensitivity to PLK1/WEE1 inhibition (Fig. 5C, D). In contrast, BRCA-WT cells showed no change or reduced sensitivity to NHEJ inhibition, highlighting distinct DNA repair pathway preferences. Importantly, siRNA knockdown of DNA-PKcs yielded similar results (Fig. 5E, F). These findings confirm that BRCA-Mut cells are highly dependent on NHEJ for survival.

### PLK1 and WEE1 inhibition suppresses tumor growth in BRCA-WT but not in BRCA-mutant xenografts

**Fig. 6:**
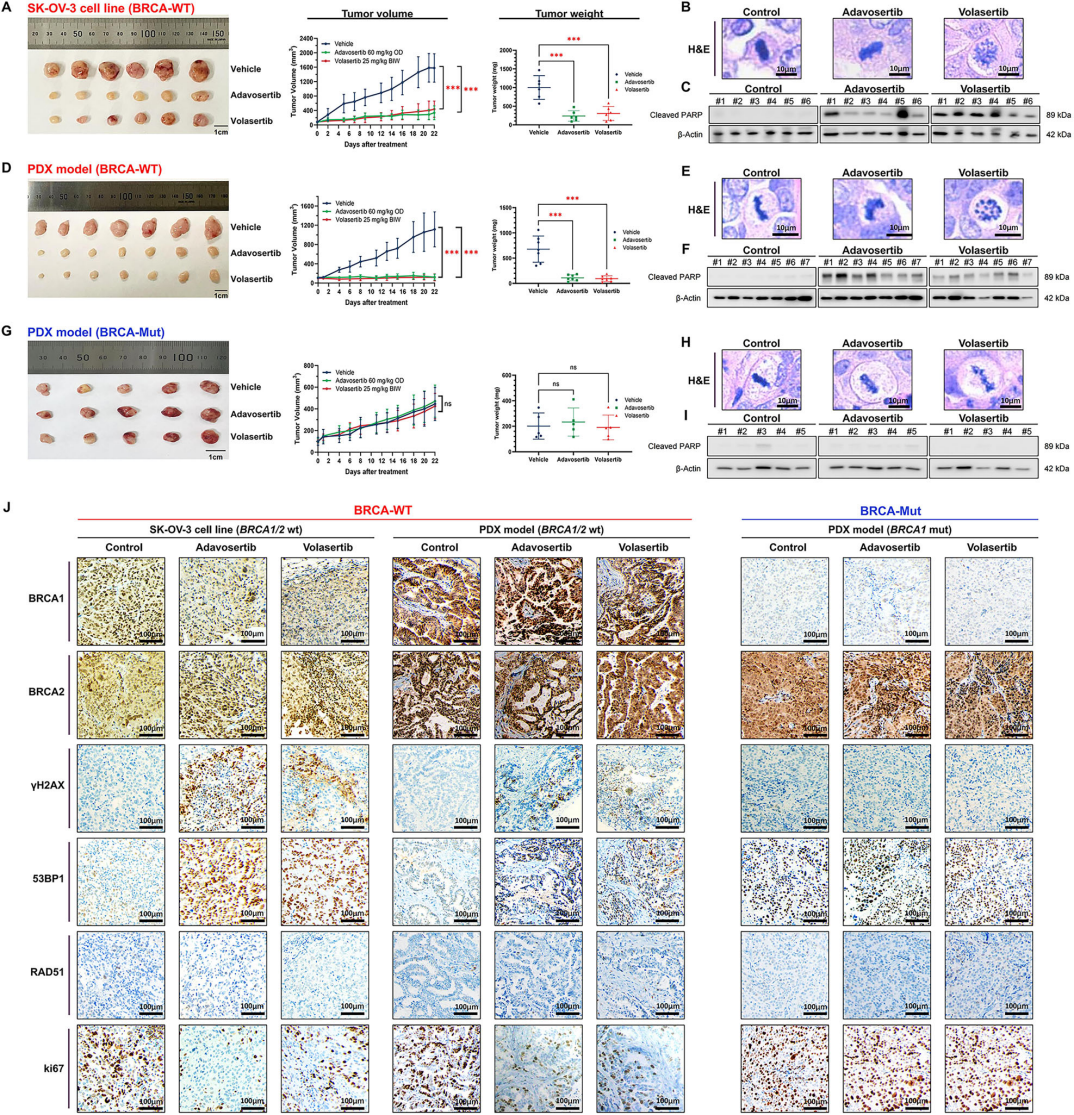
In Vivo effects of volasertib and adavosertib in xenograft and patient-derived xenograft (PDX) models of BRCA wild-type and mutant ovarian cancers. **A–C** Tumor volume measurement **A**, histological analysis of mitotic cells **B**, and cleaved PARP detection in a BRCA-WT xenograft model (SK-OV-3) treated with volasertib (60 mg/kg daily) or adavosertib (25 mg/kg twice weekly) for 3 weeks, using immunoblotting **C**. *n* = 6/group. **D–F** Tumor volume measurement **D**, histological analysis, and **E** cleaved PARP detection **F** in a BRCA-WT PDX model. *n* = 7/group. **G–I** Tumor volume **G**, mitotic analysis **H**, and cleaved PARP detection **I** in the BRCA-Mut PDX model. *n* = 5/group. **J** Immunohistochemical analysis of BRCA1, BRCA2, γ-H2AX, 53BP1, RAD51, and Ki67 expression in tumor tissues from all three models. Data are presented as mean ± SD from three independent experiments. Statistical significance: ***P < 0.001; ns, not significant using a two-way ANOVA for tumor volume comparisons and one-way ANOVA for protein quantification.

We evaluated the in vivo antitumor efficacy of PLK1 and WEE1 inhibition using three xenograft models: BRCA-WT cell line-derived model (SK-OV-3), BRCA-WT patient-derived xenograft (PDX), and BRCA-Mut PDX. The BRCA status in both PDX models was confirmed via genomic profiling (Fukushima Medical University) (Supplementary Table S3). In the SK-OV-3 model, volasertib (60 mg/kg daily) or adavosertib (25 mg/kg twice weekly) for 3 weeks significantly suppressed tumor growth without observable toxicity (Fig. 6A). H&E staining revealed abnormal mitotic figures after volasertib but not adavosertib treatment (Fig. 6B). These abnormal mitotic cells were distinguished from normal prophase cells based on established morphological criteria [45]. In addition, cleaved PARP expression was significantly upregulated after treatment with either volasertib or adavosertib (Fig. 6C). Consistent antitumor and apoptotic effects were also observed in the BRCA-WT PDX model (Fig. 6D–F). In contrast, the BRCA-Mut PDX model exhibited minimal response to either treatment, with no significant tumor reduction, mitotic abnormalities, or elevated cleaved PARP (Fig. 6G–I).

To investigate the underlying molecular mechanisms, we performed IHC assay of tumor tissues from all three models (Fig. 6J). The BRCA-Mut PDX showed BRCA1 loss, consistent with the underlying pathogenic mutation. γH2AX levels increased in both BRCA-WT models following either treatment, suggesting the accumulation of DNA damage. 53BP1 staining was detected in all models, suggesting NHEJ activity, with basal positivity in the BRCA-Mut model even without treatment, likely reflecting genomic instability due to HRD. In contrast, RAD51 was absent in either drug-treated BRCA-WT model and remained negative in BRCA-Mut PDX, consistent with the HR-deficient status. Proliferation was suppressed in BRCA-WT tumors, as shown by a marked reduction in Ki67-positive cells, and CD31 staining revealed decreased neovascularization, suggesting possible effects on the tumor microenvironment (Supplementary Fig. S6).

## Discussion

**Fig. 7:**
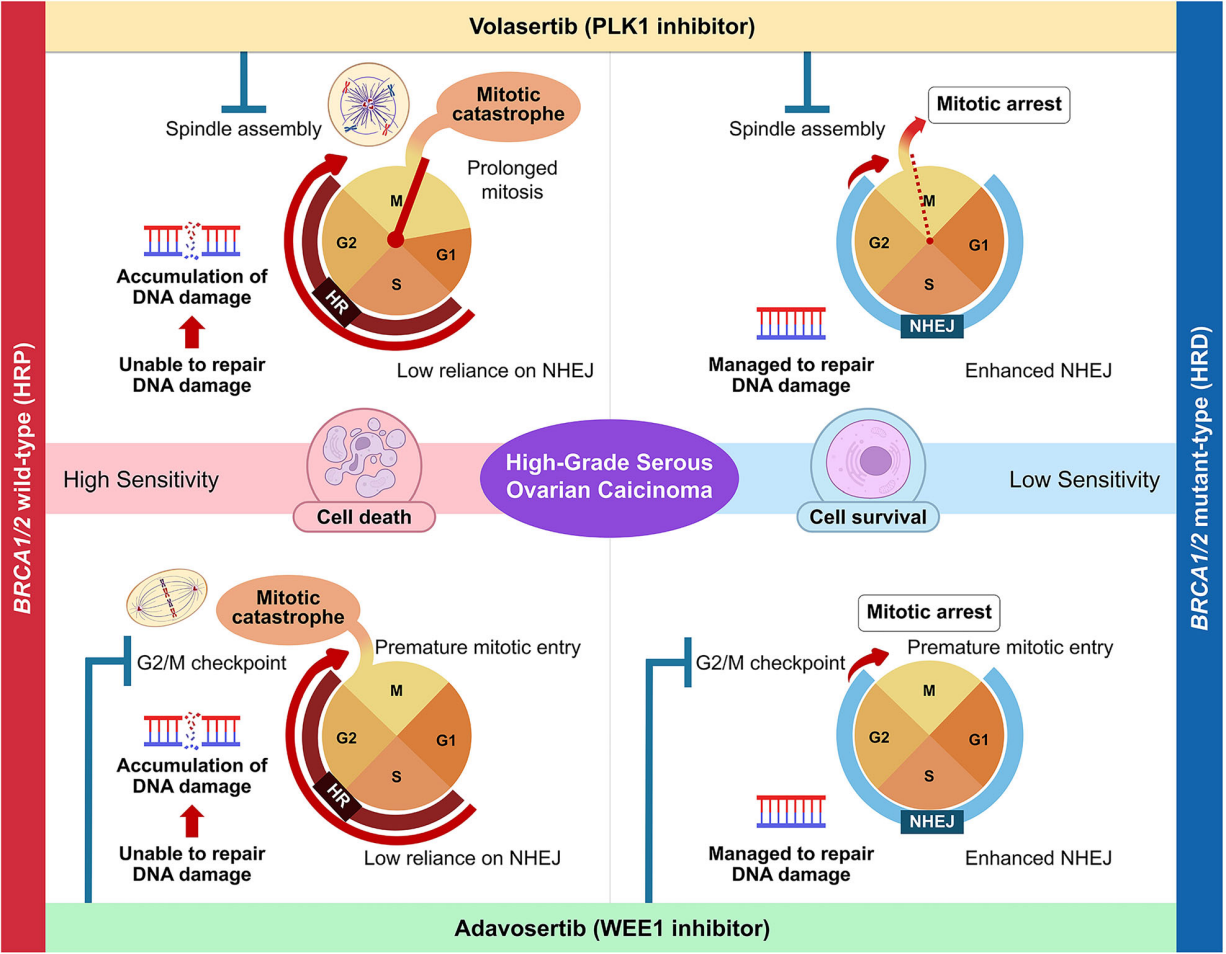
Differential mechanisms of PLK1 and WEE1 inhibitor-induced cytotoxicity in BRCA wild-type and mutant ovarian cancer cells. The schematic model illustrates how BRCA-WT and -Mut cells respond differently to PLK1 and WEE1 inhibition due to their distinct DNA repair capacities. In BRCA-WT cells, both inhibitors suppressed homologous recombination (HR), which is normally active during the S and G2 phases. PLK1 inhibition disrupts the spindle assembly checkpoint and leads to mitotic catastrophe, which is lethal when HR-mediated repair is unavailable. In contrast, WEE1 inhibition overrides the G2/M checkpoint, forcing cells to enter mitosis prematurely, with unrepaired DNA damage. BRCA-WT cells, which rely on HR for high-fidelity repair, fail to resolve mitotic damage, and undergo cell death. In BRCA-Mut cells, with HR deficient, DNA repair is maintained through enhanced non-homologous end joining (NHEJ), which is active throughout the cell cycle. BRCA-Mut cells tolerated DNA damage induced by either inhibitor and survived the treatment.

We demonstrated that BRCA-WT HGSOC cells, despite intact HR under basal conditions, were highly sensitive to PLK1 and WEE1 inhibition. This sensitivity was accompanied by impaired HR activity and a potential compensatory reliance on NHEJ, suggesting a distinct pattern of DNA repair dependency in BRCA-WT/HR-proficient cells compared to BRCA-Mut/HR-deficient cells. Previous studies indicate that up to 40% of ovarian cancer cells exhibit intrinsic defects in NHEJ pathway, highlighting that NHEJ-impairment can influence therapeutic responses [46]. BRCA-WT cells in our study expressed low DNA-PKcs with resistance to NHEJ inhibition, whereas BRCA-Mut cells displayed high DNA-PKcs and greater vulnerability to NHEJ inhibition. These findings support a low-NHEJ-reliance phenotype in BRCA-WT HGSOC. Based on these findings, we propose a conceptual framework outlining distinct mechanisms of PLK1 and WEE1 inhibition in BRCA-WT and BRCA-Mut HGSOC cells (Fig. 7).

Both PLK1 and WEE1 modulate DNA repair; PLK1 phosphorylates *BRCA1/2* and RAD51-related proteins to facilitate HR, while WEE1 maintains *BRCA2* function for RAD51 loading via CDK1 regulation [47–49]. HR is a high-fidelity repair mechanism mainly active in S and G2 phases, resolving replication-associated damage using a sister chromatid [50–53]. NHEJ operates throughout most of the cell cycle and repairs DSBs faster [54], but is error-prone and less active during mitosis [55] (Fig. 7). *BRCA1/2* play central roles in HR, and their inactivation through mutations or promoter methylation underlies HRD in HGSOC. In BRCA-WT cells (TYK-nu and SK-OV-3), PLK1 or WEE1 inhibition suppressed HR but failed to fully activate NHEJ, resulting in unrepaired DNA damage, mitotic arrest, prolonged mitosis, and apoptotic cell death. In contrast, BRCA-Mut cells (JHOS-2 and OVSAHO) maintained robust DNA repair via constitutive NHEJ, and were resistant to both inhibitors.

PLK1 plays a critical role in mitotic progression, including centrosome maturation, spindle assembly, and chromosome segregation [17, 18], and facilitates HR by phosphorylating *BRCA1*/*BRCA2* to promote RAD51 recruitment [21, 47, 48]. In BRCA-WT/HR-proficient cells, PLK1 inhibition disrupted mitotic spindle formation and activated the spindle assembly checkpoint, and prolonged mitosis (Fig. 7, upper left panel). Concomitantly, HR repair was suppressed, as evidenced by reduced RAD51 foci, leaving DSBs unresolved (Fig. 7, upper left panel). These defects caused abnormal spindle architecture, centrosomal disorganization, and increased DNA content (>4 N), leading to mitotic slippage and polyploidy, indicative of mitotic catastrophe [56]. This process ultimately led to γH2AX accumulation and cell death. In contrast, BRCA-Mut/HR-deficient cells exhibited limited sensitivity. Although PLK1 blockade induced mitotic arrest, constitutively active NHEJ, which is independent of HR and remains active throughout the G1, S, and G2 phases, enabled DNA damage repair and prevented mitotic catastrophe (Fig. 7, upper right panel). Thus, PLK1 inhibition compromises mitotic progression and disables HR support, creating synthetic lethality selectively in BRCA-WT cells.

WEE1 kinase maintains genomic stability by inhibiting CDK1 and enforcing the G2/M checkpoint, allowing time for DNA repair—particularly HR—before mitotic entry [57, 58]. In BRCA-WT/HR-proficient cells, WEE1 inhibition abrogated this checkpoint, forcing premature mitotic entry and causing replication stress with unresolved DNA damage (Fig. 7, lower left panel). HR suppression was further reinforced by direct impairment of BRCA2 phosphorylation and RAD51 loading [49], leading to defective repair, mitotic failure, and apoptotic death. In contrast, BRCA-Mut/HR-deficient cells exhibited reduced sensitivity. Although they also entered mitosis prematurely, constitutive NHEJ activity, which remains functional throughout the G1, S, and G2 phases (Fig. 7, lower right panels), allows them to mitigate DNA damage and avoid mitotic catastrophe. Thus, similar to PLK1 inhibition, WEE1 inhibition disrupts both cell cycle control and HR repair, resulting in synthetic lethality in BRCA-WT cells.

Despite their distinct roles in the cell cycle, PLK1 or WEE1 inhibition significantly disrupts mitotic progression and DNA repair. This ultimately induces mitotic catastrophe. BRCA-WT cells were more vulnerable, possibly due to their dependency on HR, while BRCA-Mut cells, more dependent on NHEJ, demonstrated reduced sensitivity. Clinically, volasertib was discontinued due to limited efficacy and toxicity, but next-generation agents such as onvansertib—an oral, selective PLK1 inhibitor—have shown encouraging preclinical and early clinical activity [59–61]. For WEE1 inhibition, adavosertib (AZD1775) has reached more advanced clinical testing. Notably, data from the EFFORT trial (NCT03579316) demonstrated a higher objective response rate (ORR) of 31% to adavosertib monotherapy in BRCA-WT patients compared to 20% in those with *BRCA* mutations [62]. Moreover, a Phase Ib study of adavosertib in combination with olaparib demonstrated activity even in tumors potentially reverting from HR-deficient to HR-proficient status [63]. These findings highlight WEE1 inhibition as a promising therapeutic approach in dynamic DNA repair contexts, and suggest that combining PLK1 or WEE1 inhibitors with NHEJ-targeting agents, such as DNA-PKcs inhibitors, may enhance the efficacy in BRCA-Mut settings. Importantly, our data suggest that their single-agent activity in HR-proficient BRCA-WT cells warrants continued clinical investigation, particularly in biomarker-stratified patient cohorts. Platinum-based chemotherapy remains the cornerstone of therapy in HGSOC, particularly for tumors with HRD, where platinum-induced DNA crosslinks are efficiently lethal. In contrast, HR-proficient tumors exhibit intrinsic or acquired platinum resistance. Therefore, PLK1 and WEE1 inhibitors may be more appropriately positioned for platinum-resistant, HR-proficient disease, supporting the development of post-platinum strategies in this setting.

In platinum-resistant, recurrent HGSOC—typically characterized by HR-proficiency— chemotherapy regimens, such as weekly paclitaxel (PTX) or gemcitabine (Gem), with or without bevacizumab, are frequently used [64, 65]. Agents targeting mitosis (PTX) or DNA synthesis (Gem) may therefore provide mechanistically compatible partners for PLK1 or WEE1 inhibitors. Both drugs are commonly administered in weekly schedules, which may allow manageable hematologic toxicity in combination settings. Accordingly, PTX + BEV or Gem-based regimens could serve as feasible backbones for further investigation in HR-proficient HGSOC. Collectively, these considerations support the development of personalized therapeutic strategies targeting DNA repair vulnerabilities, with PLK1 and WEE1 inhibitors as promising components for HGSOC treatment.

Our study has several limitations. Although we demonstrated the efficacy of PLK1 and WEE1 inhibition in BRCA-WT (HR-proficient) HGSOC models, we did not evaluate their effects in BRCA-WT tumors with functional HR deficiency or in models with acquired resistance to platinum or PARP inhibitors. Additionally, although HR activity was suppressed following PLK1 or WEE1 inhibition, molecular mechanisms, such as the role of the CHK1 pathway or BRCA phosphorylation, have not been fully elucidated. Validation in patients with HR-proficient HGSOC is necessary for its clinical translation. Whether HR proficiency serves as a tumor-agnostic biomarker and whether factors such as TP53 or RB status influence sensitivity require further investigation.

In conclusion, we identified a unique vulnerability in BRCA-WT HGSOC cells. Despite being HR-proficient under basal conditions, these cells exhibited impaired HR following PLK1 or WEE1 inhibition, leading to excessive DNA damage, mitotic arrest or prolonged mitosis, and apoptotic cell death. In contrast, BRCA-Mut cells exhibited reduced sensitivity via reliance on the NHEJ pathway. Overall, these results uncover a distinct therapeutic opportunity for HR-proficient ovarian cancers. Clinical validation is necessary to translate these findings into personalized treatment strategies, guided by DNA repair profiles.

## Materials and methods

### Gene expression and correlation analysis using GEPIA2

GEPIA2 (Gene Expression Profiling Interactive Analysis, version 2; available at [http://gepia2.cancer-pku.cn/#index]) was used to analyze RNA expression data. GEPIA2 integrates tumor and normal sample data from the Cancer Genome Atlas (TCGA) and Genotype-Tissue Expression (GTEx) projects [66]. The “Expression Analysis-Box Plots” and “Correlation Analysis” modules were employed to compare gene expression between tumor and normal tissues and to investigate the relationships between gene expression. The transcription levels were log-normalized using the log2(TPM + 1) method. A *t*-test was used to compare differences in expression between tumor and normal tissues. Correlation analyses were conducted using Spearman’s method.

### Cell culture

JHOS-2 (RRID: CVCL_4647), JHOS-3 (RRID: CVCL_4648), JHOS-4 (RRID: CVCL_4649), and OVCAR3 (RRID: CVCL_0465) cell lines were purchased from RIKEN CELL BANK (RCB; Tsukuba, Japan) [41]. KURAMOCHI (RRID: CVCL_1345), OVSAHO (RRID: CVCL_3114), OVKATE (RRID:CVCL_3110), and TYK-nu (RRID: CVCL_1776) cell lines were obtained from the Japanese Collection of Research Bioresources Cell Bank (JCRB; Osaka, Japan). SK-OV-3 (RRID: CVCL_0532) cells were obtained from American Type Culture Collection (ATCC; Manassas, VA, USA). JHOS-2, JHOS-3, JHOS-4, and OVCAR3 cells were cultured as previously reported [41]. KURAMOCHI, OVSAHO, and OVKATE cells were cultured in RPMI 1640 medium (FUJIFILM Wako, Osaka, Japan; 189-02145) supplemented with 10% fetal bovine serum (FBS). TYK-nu cells were maintained in Eagle’s minimal essential medium (FUJIFILM Wako; 051-07615) supplemented with 10% FBS. SK-OV-3 cells were cultured in McCoy’s 5 A (modified) medium (ThermoFisher Scientific, Waltham, MA, USA; 16600-082) supplemented with 10% FBS. All culture media were supplemented with 1% penicillin/streptomycin (FUJIFILM Wako; 161-23181), except for during transfection. All cell lines were cultured in a humidified incubator at 37 °C under 5% CO_2_, and were not passaged more than 15 times.

### Genomic characterization of cell lines

The mutational status of *BRCA1*/*2* and *TP53*, as well as cyclin E1 expression levels, were obtained based on data from published studies [41, 67–71] and public databases, including DepMap and ClinVar (Supplementary Table S1). Four cell lines overlapped with those used in our previous study, with three out of four cyclin E1-low cells (JHOS-2, JHOS-3, and JHOS-4) confirmed as *BRCA1*/*2* mutants and one cyclin E1-high cell (OVCAR3) confirmed as *BRCA1*/*2* wild-type (BRCA-WT). An additional four cell lines included one *BRCA1*/*2* mutant cell line (OVSAHO) and three *BRCA1*/*2* wild-type cells without cyclin E1 overexpression (TYK-nu, OVKATE, and SK-OV-3) (Supplementary Table S1). In our experiments, we included both *BRCA1*-mutant (JHOS-2) and *BRCA2*-deficient (OVSAHO) cells to represent HRD, since loss of either gene impairs HR repair and increases sensitivity to DNA-damaging agents. We used two BRCA-WT cell lines (TYK-nu and SK-OV-3) as HR-proficient models; OVKATE, although HR-proficient, was excluded because of its prolonged doubling time (96 h) and less frequently used in functional HR studies, compared with TYK-nu (doubling time: 45 h) and SK-OV-3 (doubling time: 19–44 h) [72–75].

### RNA extraction and quantitative real-time polymerase chain reaction (qRT-PCR)

Total RNA was isolated from cells using an RNeasy Mini Kit (Qiagen, Valencia, CA, USA) and reverse-transcribed to generate cDNA using ReverTra Ace qPCR RT Master Mix with gDNA Remover (TOYOBO, Osaka, Japan). qRT-PCR was performed on a LightCycler (Roche Diagnostics, Indianapolis, IN, USA) and a QuantStudio 1 Real-Time PCR System (Thermo Fisher Scientific; A40425) using the One-Step SYBR PrimeScript RT-PCR Kit (TaKaRa Bio, Tokyo, Japan). Gene expression levels were quantified using the 2^−∆∆Ct^ method and normalized to that of control genes. All experiments were performed at least three times. The primer sequences used for RT-qPCR are listed in Supplementary Table S2.

### Protein extraction and western blotting

Proteins were extracted from cells using RIPA Buffer (FUJIFILM Wako; 188-02453) supplemented with a Protease Inhibitor Cocktail (Roche; 11836153001). Notably, this protocol was applied to the cells post 72-h treatment with apoptosis inhibitors, DNA damage assays, and siRNA transfection. Cell lysates were sonicated intermittently for 10 min for complete disruption. Proteins were denatured by boiling in 4× Laemmli sample buffer (Bio-Rad, Hercules, CA, USA; #1610747) at 95 °C for 5 min. Thereafter, the proteins were separated using 4–15% Mini-PROTEAN® TGX™ Gels (Bio-Rad; #4561086) and transferred to Trans-Blot® Turbo™ Mini PVDF Transfer Packs (Bio-Rad; 1704156). Proteins were detected using Amersham ECL Select (Cytiva, Marlborough, MA, USA) and imaged using ImageQuant LAS 4000 (GE Healthcare Life Sciences, Piscataway, NJ, USA). Details of the antibodies used for the analysis are provided in Supplementary Table S2.

### Gene silencing

For gene silencing, the cells were transfected with small interfering RNAs (siRNAs) using the Lipofectamine™ RNAiMAX Transfection Reagent (Invitrogen, Thermo Fisher Scientific; 13778150), according to the RNAiMax reverse transfection protocol provided by Thermo Fisher Scientific *(ThermoFisher Scientific, 2024;* https://www.thermofisher.com/jp/ja/home/references/protocols/cell-culture/transfection-protocol/rnaimax-reverse-transfections-lipofectamine.html#procedure*)*. Specific siRNA sequences used in this study are listed in Supplementary Table S2. Notably, the final standard siRNA transfection concentration was 10 nM, except for siWEE1, which was 50 nM.

### Cell viability assay

To assess cell viability, cells were treated with volasertib (PLK1-1 inhibitor; Selleckchem, Houston, TX, USA; S2235), adavosertib (WEE1 inhibitor; Selleckchem; S1525), or NU7441 (DNA-PKcs inhibitor; Selleckchem; S2638) for 72 h. Thereafter, a Cell Counting Kit-8 solution (CCK8; FUJIFILM Wako; 341-07624) was added to each well and incubated for 2 h. Finally, the absorbance was measured at 450 nm using a BioTek microplate reader (BioTek, Winooski, VT, USA). Additionally, the 50% inhibitory concentration (IC_50_) values were calculated by fitting the data to dose-response curves using the GraphPad Prism software. All experiments were conducted in triplicate.

### Cell cycle analysis

Briefly, cells were treated with the specified inhibitor for 24 and 72 h, followed by fixation in 70% ethanol and overnight incubation at 4 °C. Thereafter, RNase A stock solution (Ribonuclease A from bovine pancreas, 0.25 mg/mL; Sigma-Aldrich, St. Louis, MO, USA; R4875) was added, followed by incubation at 37 °C for 20 min. For DNA staining, cells were incubated with 50 mg/mL of propidium iodide (PI; Sigma-Aldrich; P4170) in the dark at 4 °C for 15 min. Cell cycle analysis was performed using fluorescence-activated cell sorting (FACS) on a BD FACSCalibur™ HG Flow Cytometer (BD Biosciences, Franklin Lakes, NJ, USA), with data acquisition performed using Cell Quest Pro software v3.1 (BD Biosciences). Data analysis was conducted using FlowJo® v10.8.1 (BD Biosciences). To discriminate between G2 and M-phase populations, a dual-parameter analysis based on DNA content (7-AAD) and cyclin A2 expression was performed as previously described [41]. A gating threshold for cyclin A2 expression was established in G1-phase cells. Cells with 4 N DNA content and cyclin A2 expression above this threshold were classified as in G2, whereas those below this threshold were classified as in M-phase. Compensation was adjusted using a FITC-conjugated mouse IgG1 isotype control (Beckman Colter, Brea, CA, USA, A07795). Flow cytometry was performed using a CytoFLEX instrument (Beckman Colter) according to standard dual-color protocols. The experiments were repeated three times.

### Immunofluorescence assay

Briefly, cells seeded on coverslips were fixed with 4% paraformaldehyde (FUJIFILM Wako; 163-20145) at 22 °C for 10 min. After rinsing twice with 0.05% Tween 20 (Sigma-Aldrich; P7949) in PBS and permeabilizing with 0.3% Triton X-100 (Sigma-Aldrich; T9284) in PBS for 10 min, the cells were blocked with PBS containing 3% BSA (Gibco, Thermo Fisher Scientific; 15260037) for 40 min. Thereafter, the cells were incubated overnight at 4 °C with primary antibodies diluted in 3% BSA in a moist chamber (antibodies listed in Supplementary Table S2). After rinsing twice with 0.05% Tween 20 in PBS, the cells were incubated in the dark with secondary antibodies diluted in 3% BSA for 1 h. For F-actin staining, the cells were incubated in the dark with rhodamine-conjugated phalloidin (Cat. #PHDR1; Cytoskeleton, Inc., Denver, CO, USA) at room temperature for 30 min. The cells were stained with DAPI (0.5 µg/ml; Sigma-Aldrich; D9542) for 5 min. After additional washing, slides were mounted with ProLong Gold (Life Technologies, Thermo Fisher Scientific; P36930) and examined under an LSM700 confocal microscope (Carl Zeiss, Le Pecq, France). Foci quantification was performed using the ImageJ software (NIH, Bethesda, Maryland, USA), with over 100 cells counted per experiment. Notably, γH2AX foci, indicative of DNA lesions, were distinguished from pan-nuclear staining, which is a diffuse pattern of γH2AX not included in foci counts [76]. To assess HR proficiency, cells were treated with a DNA-damaging agent (cisplatin at 20 µM) to induce HR activity. RAD51 foci were detected using immunofluorescence staining as described above. HR activity was quantified based on the percentage of cells containing ≥ 5 discrete RAD51 foci, with over 50 cells analyzed per sample. Error bars represent standard error of mean (SEM) derived from three independent experiments. For morphological analysis, the cells were fixed using paraformaldehyde and examined under an optical microscope (Olympus ckx53, Olympus, Tokyo, Japan) equipped with ×40 objective lens and ×10 eyepiece.

### Xenograft and PDX models

Briefly, 6-week-old female BALB/cSlc-nu/nu mice (Japan SLC, Shizuoka, Japan) were subcutaneously injected with SK-OV-3 cells (1 × 10⁷ cells in 200 µL PBS). Patient-derived tumor tissues (F-PDX ID: F_PDX_0000134 and F_PDX_000007; Fukushima Medical University, Japan) were implanted into 6-week-old female NOD.Cg-Prkdc<scid> Il2rg<tm1Sug > /Jic (NOG) mice (In Vivo Science Inc., Tokyo, Japan). All experiments were conducted under pathogen-free (SPF) conditions at an SPF animal facility. Mice were housed in individually ventilated metal cages (three mice per cage) under a 12 h/12 h light/dark cycle at a controlled temperature of 22 ± 2 °C and 50 ± 10% humidity. Once the tumors reached a volume of approximately 100–200 mm^3^, the mice were randomized and treated with vehicle, volasertib (25 mg/kg, twice weekly), or adavosertib (60 mg/kg, once daily) for 3 weeks. Blinding was not performed in the animal studies. Adavosertib was freshly prepared in 0.5% methylcellulose (FUJIFILM Wako, Japan) and volasertib was prepared weekly in corn oil (FUJIFILM Wako) to maintain stability and cover the scheduled dosing. Tumor volumes were measured three times per week using the following formula: (4π/3) × (width/2)² × (length/2). At the end of the experiment, the mice were euthanized and the tumors were resected and bisected. One portion was snap-frozen for protein extraction and the other was fixed in 10% formalin neutral buffer solution (FUJIFILM Wako; 062-01661) for histological and immunohistochemical analyses. Clinical and genomic information on the PDX models is provided in Supplementary Table S3. All animal procedures were approved by the Institutional Animal Care and Use Committee of the University of Tokyo (Approval number: A24M0450).

### Immunohistochemistry

Immunohistochemical (IHC) assay was performed on tumor samples collected from the xenograft models. Briefly, fixed tissues were dehydrated using a graded ethanol and xylene series, embedded in paraffin, cut into 3-µm thick sections for H&E-staining and IHC assay. For IHC assay, the sections were deparaffinized and incubated with 3% hydrogen peroxide at 22 °C for 5 min to block endogenous peroxidase activity. Antigen retrieval was performed in a citric acid-based Antigen Unmasking Solution (H-3300; Vector Laboratories, CA, USA) at 120 °C for 10 min using a pressure cooker. After cooling, nonspecific binding was blocked by incubation with ImmunoBlock (CTKN001; KAC, Kyoto, Japan) for 5 min at RT. Thereafter, the sections were incubated overnight at 4 °C with specific primary antibodies (Supplementary Table S2). After washing with tris-buffered saline (TBS), the sections were incubated with a secondary antibody (Simple Stain MAX-PO (R), 424142, Nichirei, Tokyo, Japan) at 37 °C for 30 min. Signals were developed using the ImmPACT DAB peroxidase substrate (SK-4105; Vector Laboratories, Burlingame, CA, USA), and the nuclei were counterstained with hematoxylin. Images were captured using an Olympus DP74 camera (Olympus) and Olympus BX53 microscope equipped with a ×20 objective lens (Olympus). Mitotic cells were visualized in hematoxylin and eosin (H&E)-stained sections using a ×40 objective lens. Images were acquired and analyzed using Olympus cellSens Standard 3.2 software.

### Statistical analyses and graphic representations

All statistical analyses and graph generation were performed using GraphPad Prism software version 9.5.1. Data are presented as mean ± SD. Assumptions of normality and equal variance were assessed prior to statistical testing. For normally distributed data, comparisons between two groups were conducted using the unpaired two-tailed Student’s t-test, and among three or more groups using one-way analysis of variance (ANOVA) followed by Tukey’s multiple comparisons test. For non-normally distributed data, the Kruskal-Wallis H-test was applied. All experiments were performed in triplicate unless otherwise noted. Statistical significance was set at *P* < 0.05 (*p < 0.05, **p < 0.01, ***p < 0.001).

## Supplementary information

Supplementary Figures and Tables

Uncropped western blots

## Data Availability

The data that support the findings of this study are available from the corresponding author upon reasonable request.
