# Supplementary material for: PLK1 or WEE1 inhibition targets homologous recombination repair proficiency in BRCA1/2 wild-type high-grade serous ovarian cancer
Source: Cell Death Dis. 2025 Dec 7;16(1):905. doi: 10.1038/s41419-025-08324-2 (PMC12727844; doi:10.1038/s41419-025-08324-2)
Supplement: Supplementary file 1 — Supplementary Figures and Tables [file 41419_2025_8324_MOESM1_ESM.docx]

**Supplementary Information**

**PLK1 or WEE1 inhibition targets** **homologous recombination proficiency in *BRCA1*/*2* wild-type high-grade serous ovarian cancer**

Qian Xi^1,2^, Akiko Kunita^3, 4^*, Miho Ogawa^3^, Masanori Kawakami^5^, Mirei Ka^1^, Saeko Nagai^6^, Anh Quynh Duong^6^, Ayumi Taguchi^6^, Kousuke Watanabe^3^, Tomohiko Fukuda^7^, Kenbun Sone^6^, Aya Shinozaki-Ushiku^1,4^, Tetsuo Ushiku^4^, Yasushi Hirota^6^, Hidenori Kage^5^, Kazuhiro Katayama^8^, Katsutoshi Oda^1^*

^1^Division of Integrative Genomics, Graduate School of Medicine, The University of Tokyo, Tokyo, Japan

^2^Department of Gynecological Oncology, Sun Yat-sen University Cancer Center, Guangzhou, China.

^3^Next-Generation Precision Medicine Development Laboratory, Graduate School of Medicine, The University of Tokyo, Tokyo, Japan

^4^Department of Pathology, Graduate School of Medicine, The University of Tokyo, Tokyo, Japan

^5^Department of Respiratory Medicine, Graduate School of Medicine, The University of Tokyo, Tokyo, Japan

^6^Department of Obstetrics and Gynecology, Graduate School of Medicine, The University of Tokyo, Tokyo, Japan

^7^Department of Obstetrics and Gynecology, Faculty of Medicine, University of Yamanashi, Yamanashi, Japan

^8^Laboratory of Molecular Targeted Therapeutics, School of Pharmacy, Nihon University, Chiba, Japan

***Correspondence to:** Akiko Kunita, Email: kunita@m.u-tokyo.ac.jp; Katsutoshi Oda, Email: odak@g.ecc.u-tokyo.ac.jp

This PDF file includes:

Supplementary Figures S1 to S6

Supplementary Tables S1 to S3


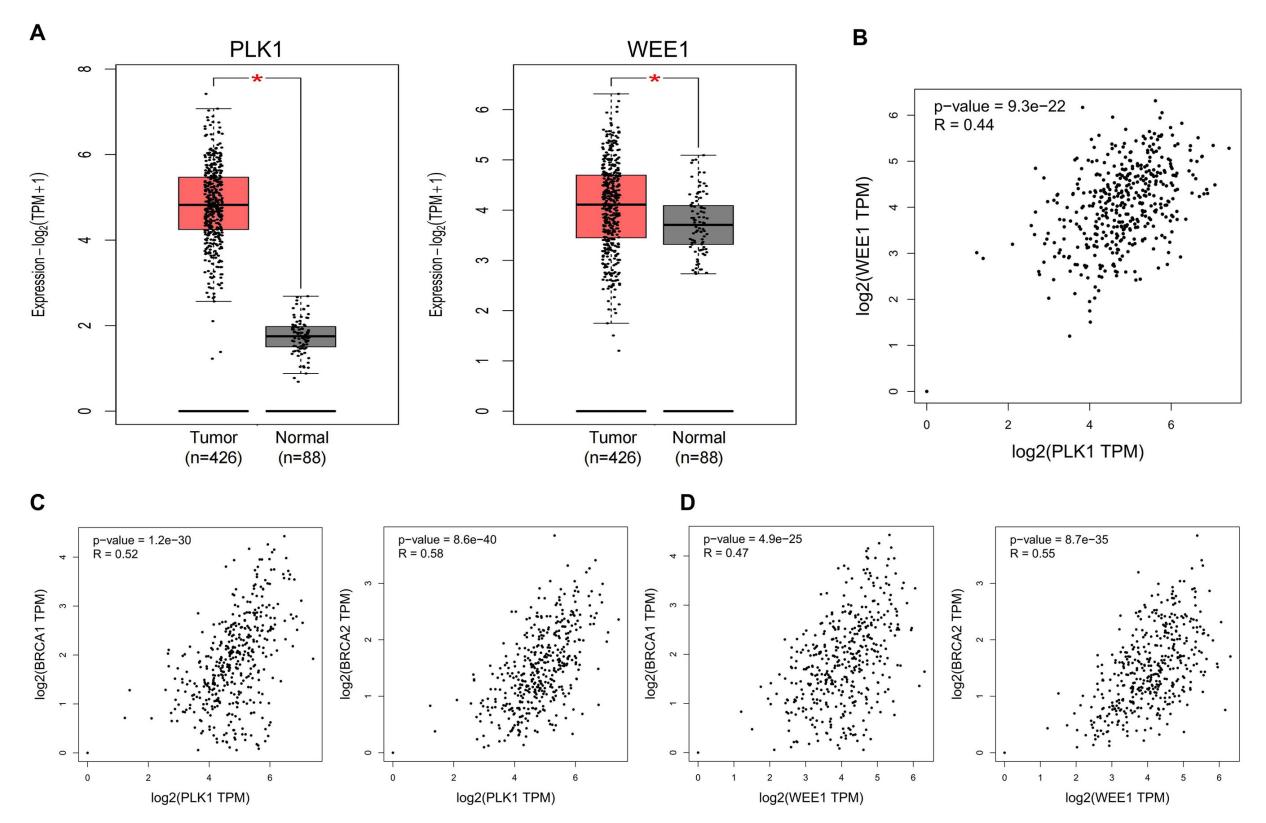


**Supplementary Figure S1. Expression and correlation analysis of *PLK1*, *WEE1*, and *BRCA1/2* genes in ovarian cancer.** (**A**) Elevated expression (**P* < 0.05) of *PLK1* and *WEE1* in ovarian cancer tissues (n = 426) compared with that in normal ovarian tissues (n = 88). (**B**) A positive correlation was observed between *PLK1* and *WEE1* expression in ovarian cancer. (**C, D**) A significant positive linear correlation was observed between the expression levels of *PLK1*, *WEE1*, and *BRCA1/2* in ovarian cancer. Correlations were analyzed using Spearman's method. Data derived from TCGA (ovarian cancer tissues) and GTEx (normal ovarian tissues) databases are presented on the GEPIA2 platform. The transcription levels were log-normalized using the log2(TPM+1) method.
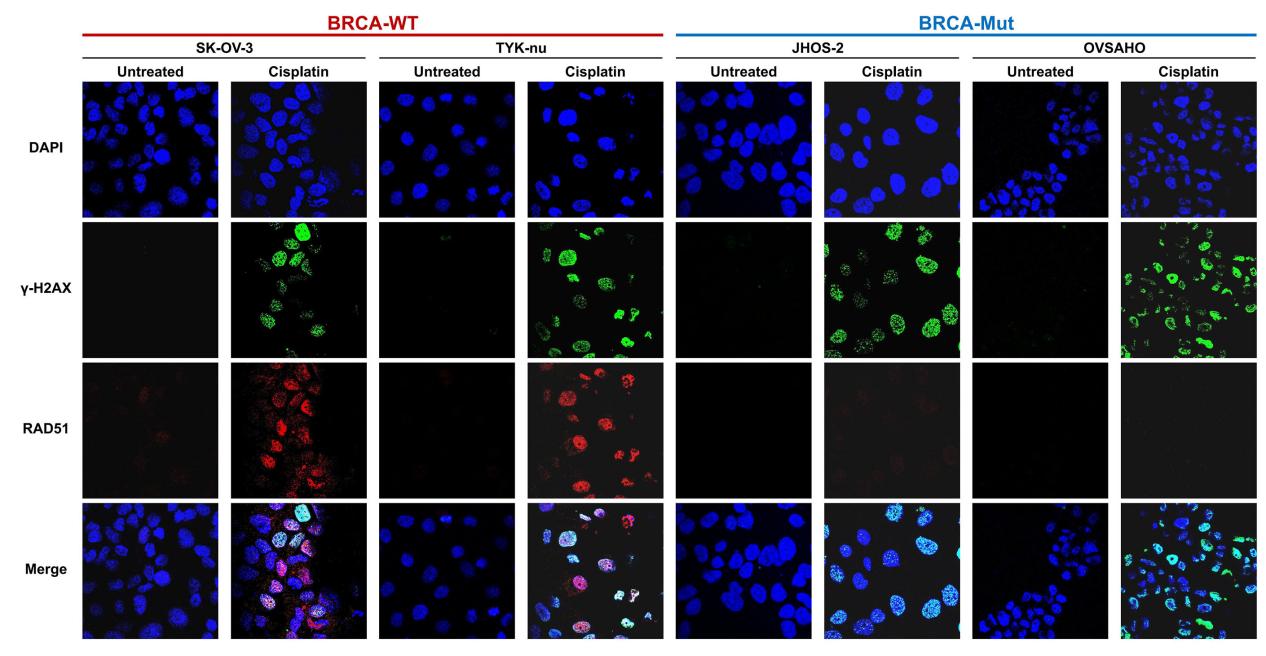


**Supplementary Figure S2. RAD51 assay was performed to evaluate homologous recombination status in ovarian cancer cells.** Immunofluorescence analysis of BRCA1/2 wild-type ovarian cancer cell lines. Cells were treated with cisplatin (20 μM, 24 h) to induce DNA damage and stained with antibodies specific to RAD51 (representing homologous recombination activity) and γH2AX (a marker of DNA double-strand breaks).


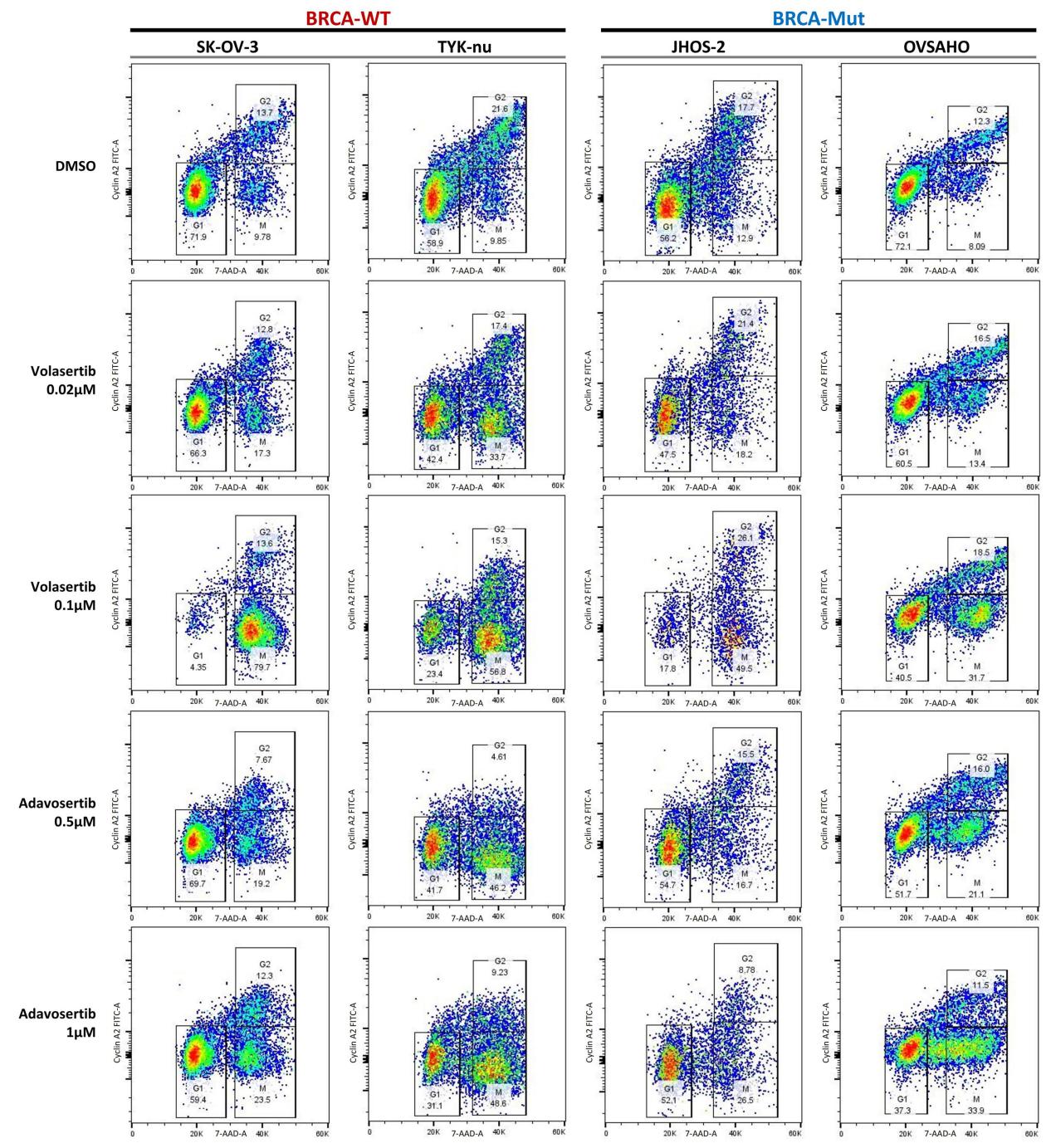


**Supplementary Figure S3. Cell cycle phase analysis using cyclin A2 and 7-AAD dual staining.** BRCA-wild type (SK-OV-3 and TYK-nu) and BRCA-mutant (JHOS-2 and OVSAHO) ovarian cancer cell lines were treated with control, volasertib, or adavosertib for 24 h, followed by immunostaining with cyclin A2 and 7-AAD to distinguish the cell cycle phases. Based on cyclin A2 expression and DNA content, the proportions of cells in the G1, S, M, and G2 phases were determined. Sub-G1 and other undefined populations were excluded from analysis.


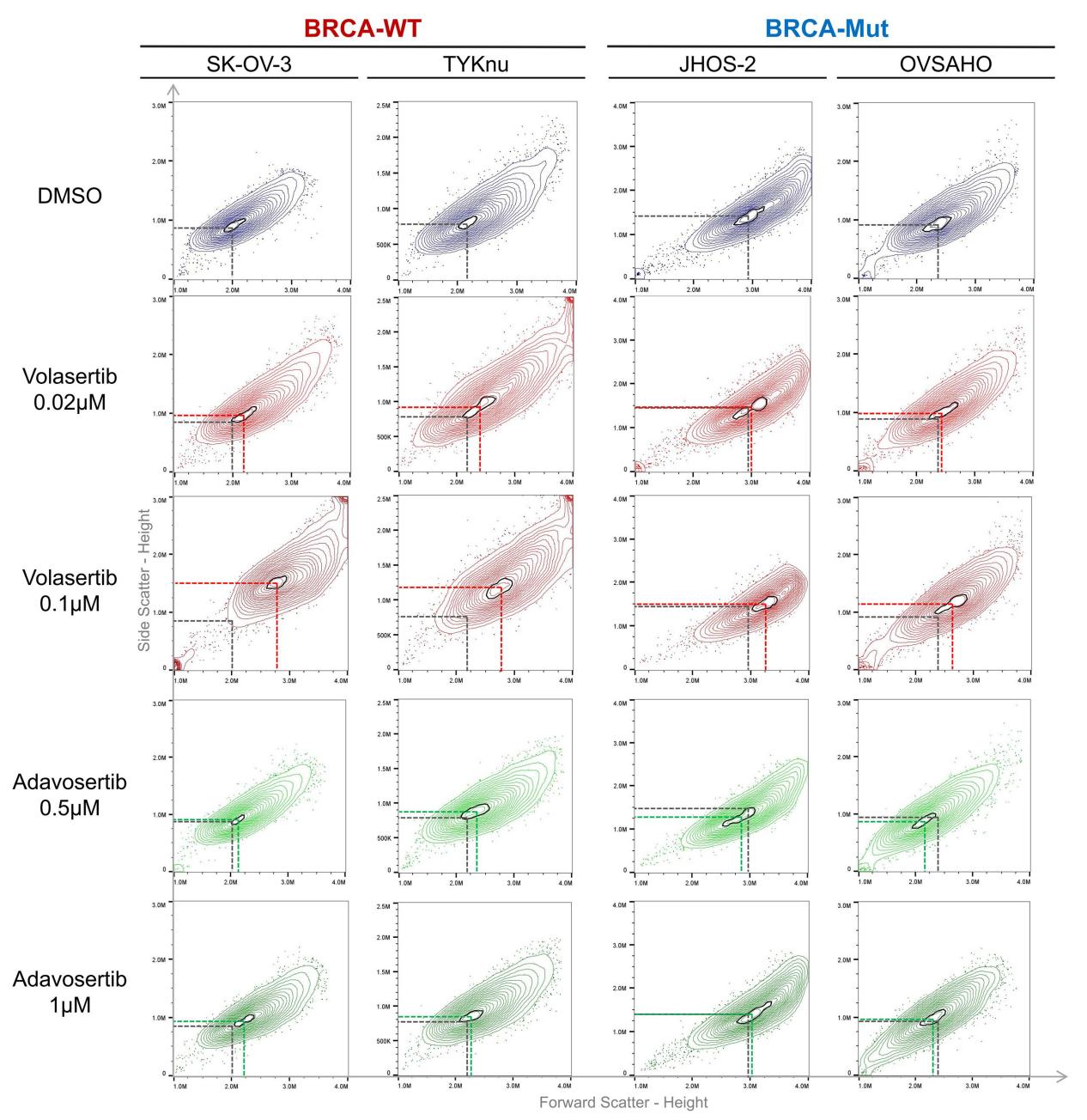


**Supplementary Figure S4. Flow cytometry-based assessment of cell size following treatment with volasertib and adavosertib.** Forward scatter height (FSC-H) versus side scatter height (SSC-H) plots were used to assess the relative cell sizes of *BRCA1/2* wild-type (SK-OV-3, TYK-nu) and mutant (JHOS-2, OVSAHO) cells after treatment with volasertib and adavosertib. An upward and rightward shift indicated an increase in cell size.


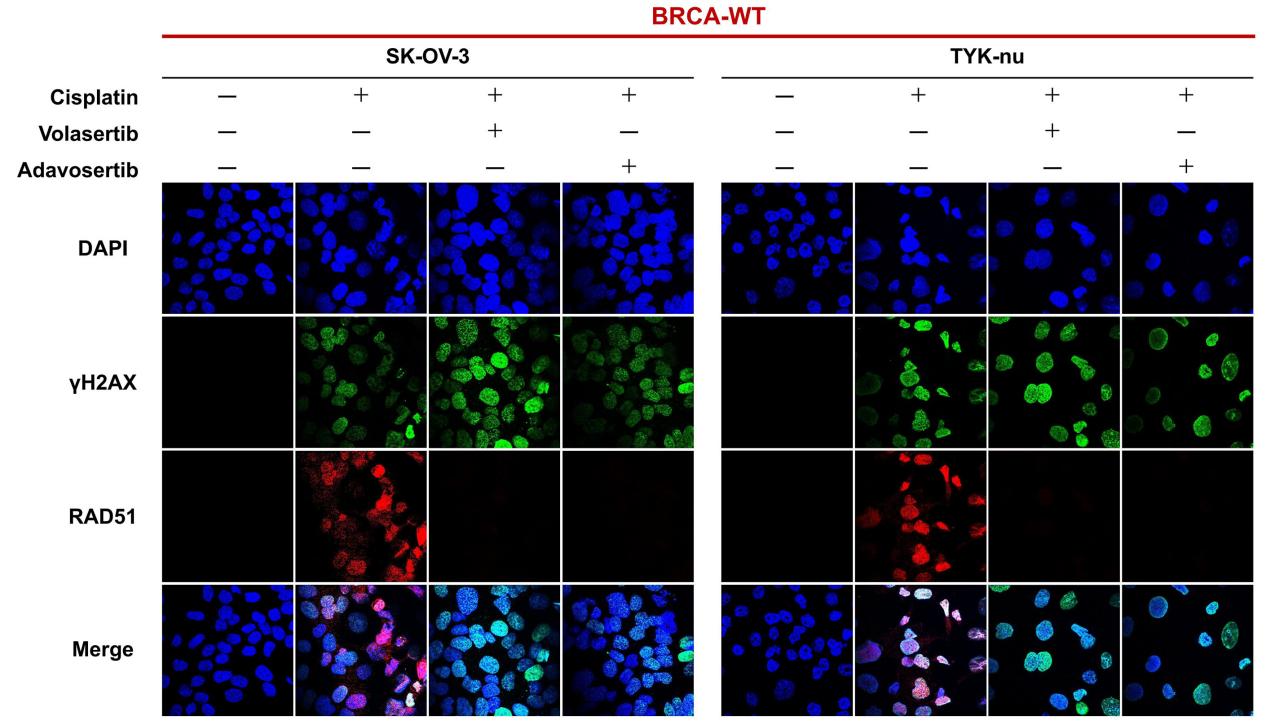


**Supplementary Figure S5. Inactivation of the homologous recombination pathway in *BRCA1/2* wild-type cells following volasertib and adavosertib treatment.** Immunofluorescence analysis of *BRCA1/2* wild-type ovarian cancer cell lines (SK-OV-3 and TYK-nu). Cells were pre-treated with cisplatin (20 μM, 24 h) to induce DNA damage, followed by an additional 24-h treatment with adavosertib or volasertib. The cells were stained with antibodies specific to RAD51 (represents homologous recombination [HR] activity) and γH2AX (a marker of DNA double-strand breaks).


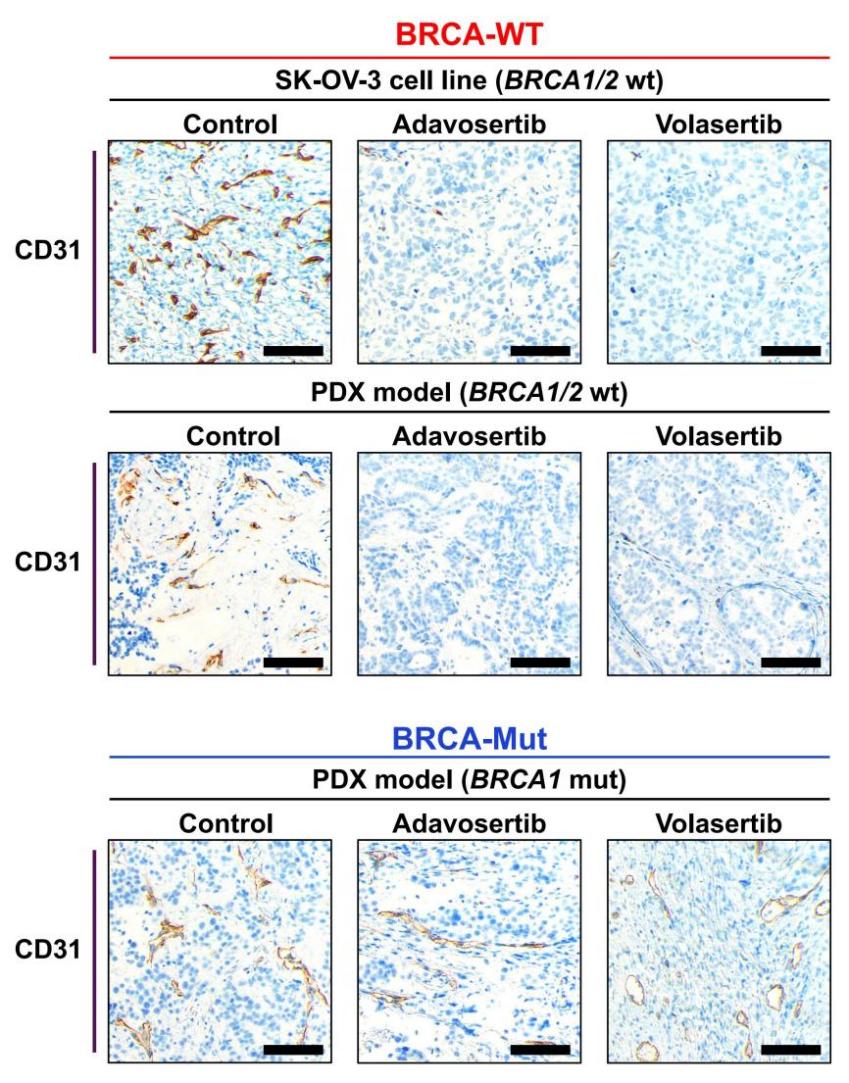


**Supplementary Figure S6. CD31 immunohistochemical analysis of tumor vasculature in BRCA wild-type and BRCA-mutant models.** Tumor tissues from xenografts and patient-derived xenograft (PDX) models treated with volasertib or adavosertib were subjected to CD31 staining to assess neovascularization. Representative images are shown for BRCA-WT (SK-OV-3 and BRCA-WT PDX) and BRCA-mutant PDX tumors. Scale bar = 100 µm.

**Supplementary Table S1. Gene Status in Ovarian Cancer Cell Lines.**

| **Cell lines** | BRCA1 status | BRCA2 status | CCNE1 expression | TP53 status |
| --- | --- | --- | --- | --- |
|  |  |  |  |  |
| JHOS-2 | c.1A>G (p.M1?); Pathogenic | - | Low | c.767_782+4delTGACCTGGAGTCTTCCAGTG; Pathogenic/Likely pathogenic |
| JHOS-3 | c.3603_3616del (p.Y1202QfsTer12); Pathogenic | - | Low | c.783-1G>T; Pathogenic/Likely pathogenic |
| JHOS-4 | c.5278-1G>A; Pathogenic | - | Low | c.T440G (p.Val147Gly); Likely pathogenic |
| OVSAHO | - | homozygous deletion; Pathogenic | Low | c.1024C>T (p.Arg342Ter); Pathogenic |
| OVKATE | - | - | Low | c.844C>T (p.Arg282Trp); Pathogenic/Likely pathogenic |
| TYK-nu | - | - | Low | c.524G>A (p.Arg175His); Pathogenic |
| OVCAR3 | - | - | High | c.743G>A (p.Arg248Gln); Pathogenic |
| SK-OV-3 | - | - | Low | c.267delC (p.Ser90Profs*33); Pathogenic |

**Supplementary Table S2. Used Antibodies, Primers, and siRNAs.**

| **Primers** | | | | |
| --- | --- | --- | --- | --- |
| **Name** | **Forward Sequence** | | | **Reverse Sequence** |
| PLK1 | AACACGCCTCATCCTCTACAAT | | | AGGAGGGTGATCTTCTTCATCA |
| WEE1 | GACGAAGATGATTGGGCATCC | | | TGGACTGGAGATCCTTGTTACA |
| β-actin | CACACTGTGCCCATCTACGA | | | CTCCTTAATGTCACGCACGA |
| DNA-PKcs | GGCCATGACGAGAGGGAACAC | | | TGAGAGCTGGCGAAGTGGGAGC |
| **Primary Antibodies** | | | | |
| **Name** | **RRID** | **Application** | **Working concentration** | **Company** |
| anti-PLK (F-8) (SC-17783) | AB_628157 | WB | 1:200 | Santa Cruz Biotechnology |
| anti-Wee1 (B-11) (SC-5285) | AB_628447 | WB | 1:200 | Santa Cruz Biotechnology |
| anti-Caspase-3 (#9662) | AB_331439 | WB | 1:1000 | Cell Signaling Technology |
| anti-Cleaved Caspase-3 (Asp175) (5A1E) (#9664) | AB_2070042 | WB | 1:1000 | Cell Signaling Technology |
| anti-PARP (#9542) | AB_2160739 | WB | 1:1000 | Cell Signaling Technology |
| anti-Cleaved PARP (Asp214) (D64E10) XP® (#5625) | AB_10699459 | WB | 1:1000 | Cell Signaling Technology |
| anti-Histone H2A.X (ab11175) | AB_297814 | WB | 1:1000 | Abcam |
| anti-Phospho-Histone H2A.X (Ser139) (#2577) | AB_2118010 | WB | 1:1000 | Cell Signaling Technology |
| anti-DNA PKcs (18-2) (ab44815) | AB_731982 | WB | 1:1000 | Abcam |
| anti-Chk1 (2G1D5) (#2360) | AB_2080320 | WB | 1:1000 | Cell Signaling Technology |
| anti-Chk2 (1C12) (#3440) | AB_2229490 | WB | 1:1000 | Cell Signaling Technology |
| anti-Rad51 [EPR4030(3)] | AB_2722613 | WB | 1:1000 | Abcam |
| anti-DNA PKcs [18-2] (ab44815) | AB_731982 | WB | 1:1000 | Abcam |
| anti-β-actin (A2228) | AB_476697 | WB | 1:10000 | Sigma-Aldrich |
| anti-Phospho-Histone H2A.X (Ser139) (05-636) | AB_309864 | IF | 1:1000 | Merck Millipore |
| anti-RAD51 (clone 1M8) (ZRB1492) | - | IF | 1:1000 | Merck ZooMAb |
| anti-53BP1 (clone 1H16) (ZRB1153) | - | IF | 1:100 | Merck ZooMAb |
| anti-α-Tubulin (clone DM1A) (T6199) | AB_477583 | IF | 1:1000 | Sigma-Aldrich |
| anti-Pericentrin (ab4448) | AB_304461 | IF | 1:1000 | Abcam |
| anti-Ki-67 (D3B5) (#9129) | AB_2687446 | IHC | 1:1000 | Cell Signaling Technology |
| anti-Phospho-Histone H2A.X (Ser139) (20E3) (#9718) | AB_2118009 | IHC | 1:1000 | Cell Signaling Technology |
| anti-BRCA1 (ab213929) | AB_2893209 | IHC | 1:300 | Abcam |
| anti-BRCA2 (ab216972) | - | IHC | 1:300 | Abcam |
| anti-53BP1 (ab175933) | AB_2890610 | IHC | 1:50 | Abcam |
| anti-Rad51 (ab133534) | AB_2722613 | IHC | 1:50 | Abcam |
| anti-CD31 (PECAM-1) (D8V9E) (#77699) | AB_2722705 | IHC | 1:1000 | Cell Signaling Technology |
| **Secondary Antibodies** | | | | |
| **Name** | **RRID** | **Application** | **Working concentration** | **Company** |
| anti-mouse IgG,HRP-linked antibody (#7076) | AB_330924 | WB | 1:3000 | Cell Signaling Technology |
| anti-rabbit IgG, HRP-linked antibody (#7074) | AB_2099233 | WB | 1:3000 | Cell Signaling Technology |
| goat anti-rabbit Alexa Fluor® 568 (ab175471) | AB_2576207 | IF | 1:1000 | Abcam |
| donkey anti-mouse Alexa Fluor® 488 (ab150105) | AB_2732856 | IF | 1:1000 | Abcam |
| **siRNAs** | | | | |
| **Name** | **Sense Sequence** | | | **Antisense Sequence** |
| siPLK1#1 | CCGGAUCAAGAAGAAUGAAUA | | | UAUUCAUUCUUCUUGAUCCGG |
| siPLK1#2 | UGAAGAAGAUCACCCUCCUUA [dT][dT] | | | UAAGGAGGGUGAUCUUCUUCA [dT][dT] |
| siWEE1#1 | GCAUUCUCAUGUAGUUCGAUU | | | AAUCGAACUACAUGAGAAUGC |
| siWEE1#2 | AAUAUGAAGUCCCGGUAUA | | | UAUACCGGGACUUCAUAUU |
| siDNA-PKcs#1 | GAACAUGGCAGGAGAGAAU [dT][dT] | | | AUUCUCUCCUGCCAUGUUC [dT][dT] |
| siDNA-PKcs#2 | CUUUAUGGUGGCCAUGGAG [dT][dT] | | | CUCCAUGGCCACCAUAAAG [dT][dT] |
| MISSION® siRNA Universal Negative Control #1 | SIC001, Sigma-Aldrich | | | |
| * WB, Western Blotting; IF: Immunofluorescence; IHC: Immunohistochemistry. | | | | |

**Supplementary Table S3. Clinical and genetic profiles of F-PDX® ovarian cancer models established in the Fukushima project.**

| No. | PDX classification | F-PDX ID | F-PDX name | Tissue origin | Sex | Age (years) | Country | Histopathological findings | *BRCA1* status | *BRCA2* status |
| --- | --- | --- | --- | --- | --- | --- | --- | --- | --- | --- |
| 1 | *BRCA1/2* wild-type | F_PDX_0000134 | DOVA017 | Metastatic site; F-PDO | Female | 67 | Japan | Serous papillary adenocarcinoma | wild type | wild type |
| 2 | *BRCA1/2* mutant | F_PDX_000007 | DOVA007 | Metastatic site; Recurrent tumor | Female | 70 | Japan | Serous papillary adenocarcinoma | Gln867Ter, Pathogenic | wild type |
