# Supplementary figures and images for: PLK1 or WEE1 inhibition targets homologous recombination repair proficiency in BRCA1/2 wild-type high-grade serous ovarian cancer
Source: Cell Death Dis. 2025 Dec 7;16(1):905. doi: 10.1038/s41419-025-08324-2 (PMC12727844; doi:10.1038/s41419-025-08324-2)

Fig. 1C

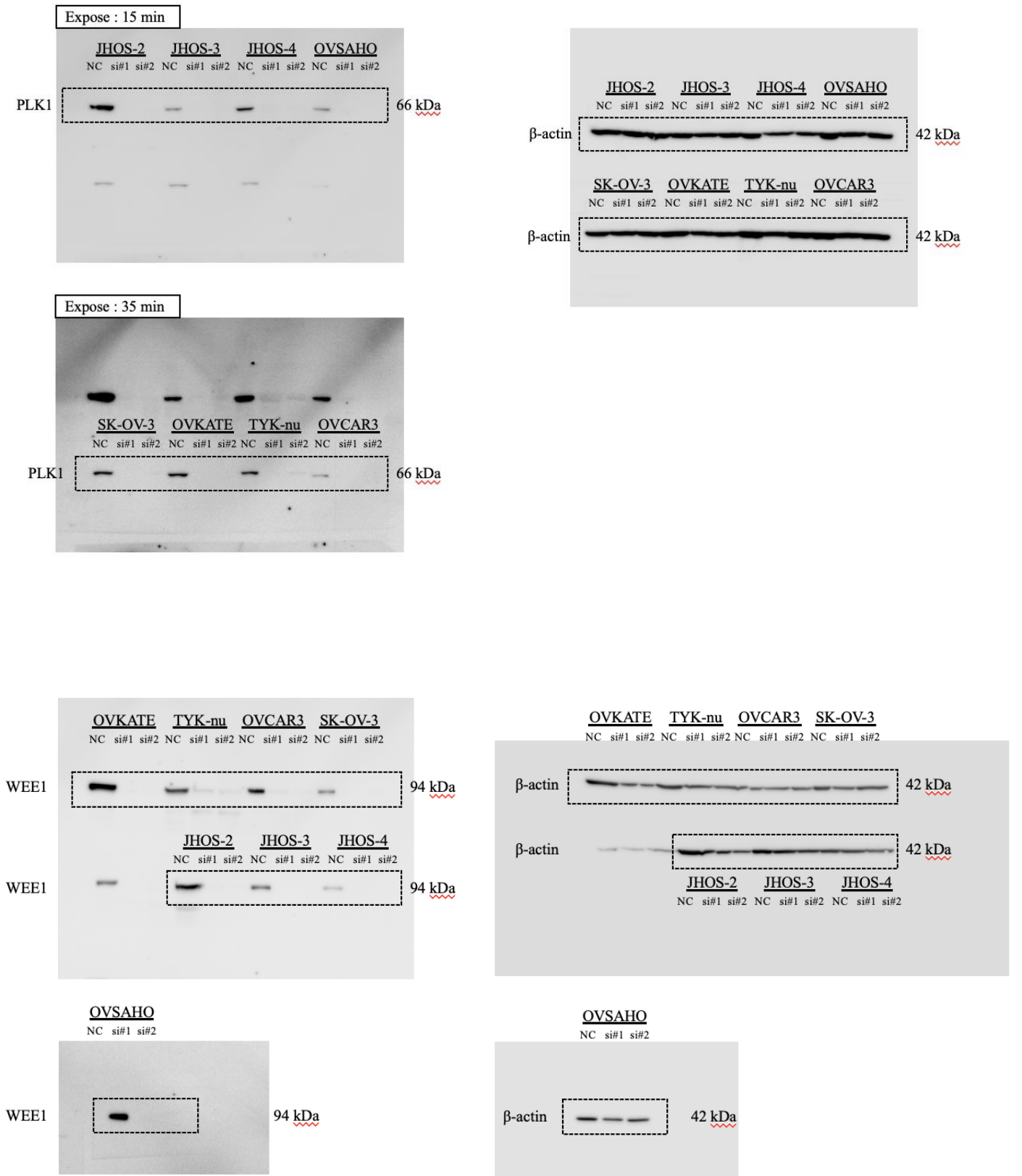

Fig. 4F

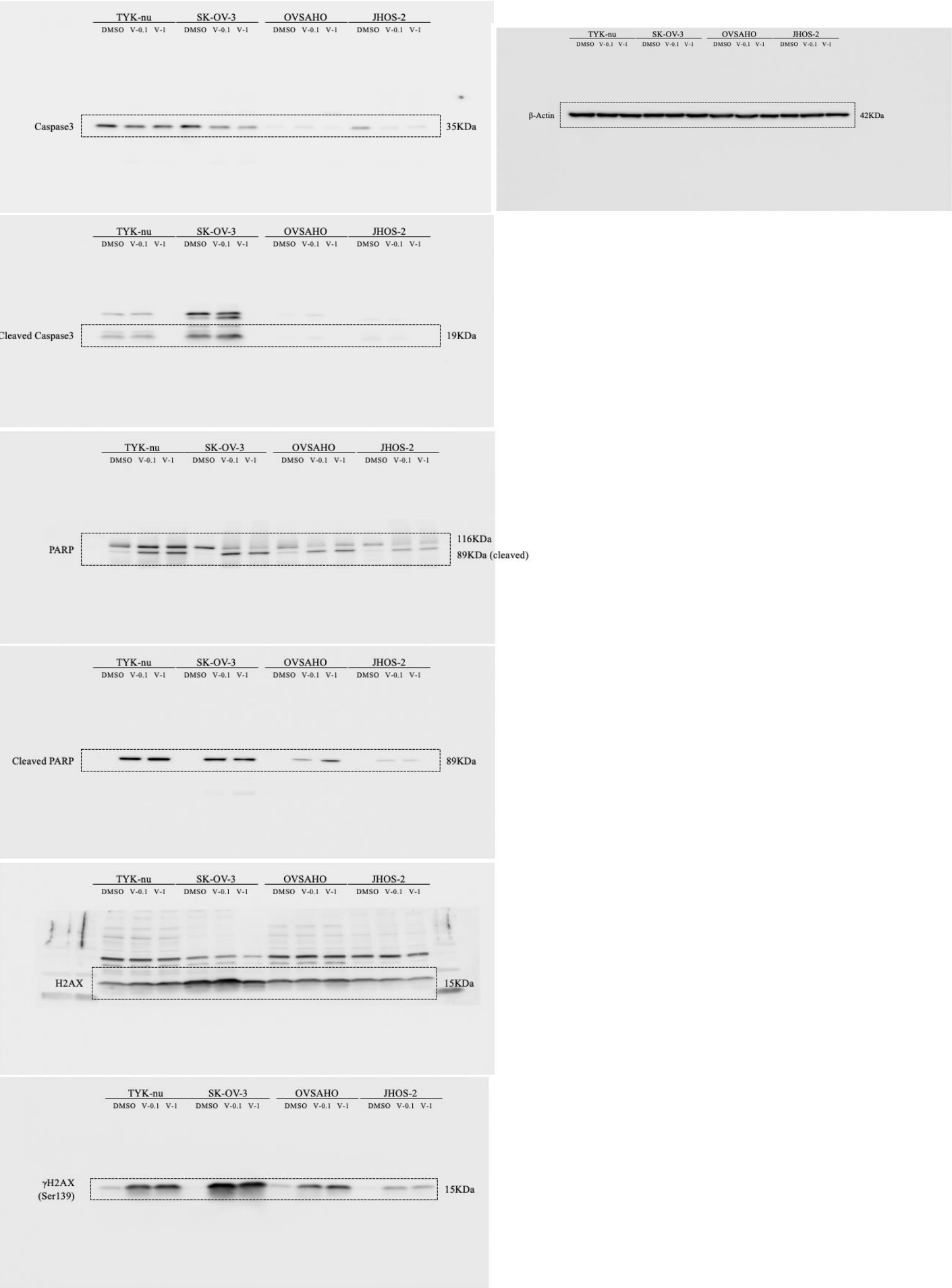

Fig. 4G

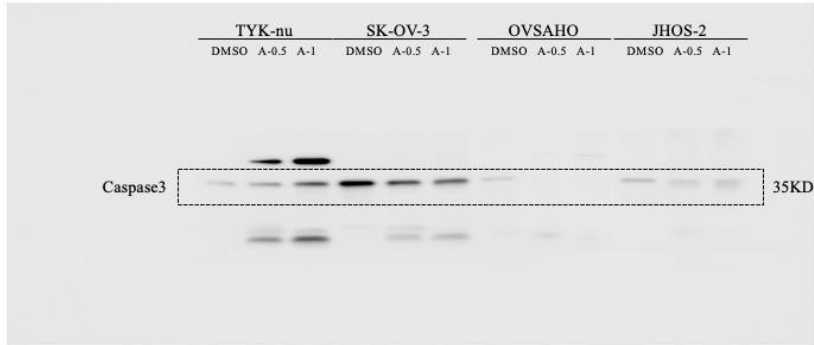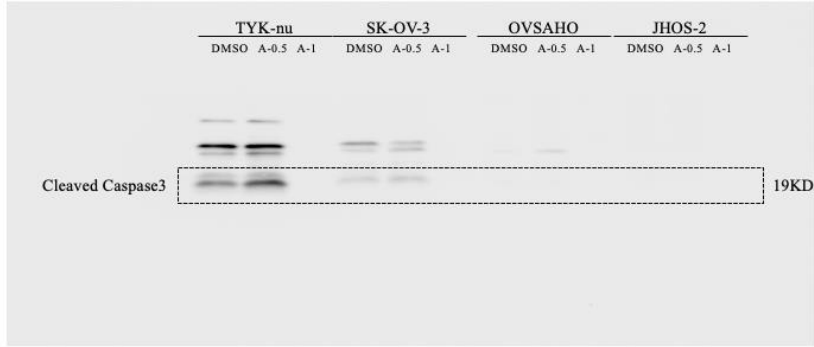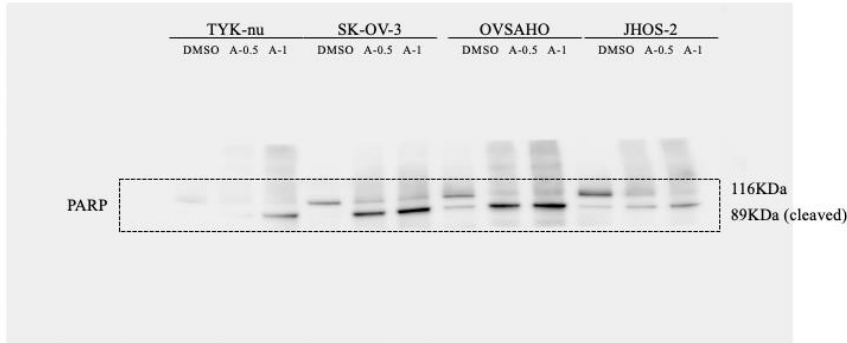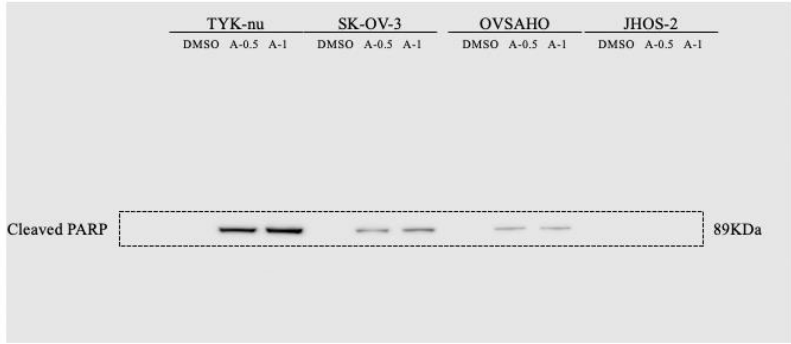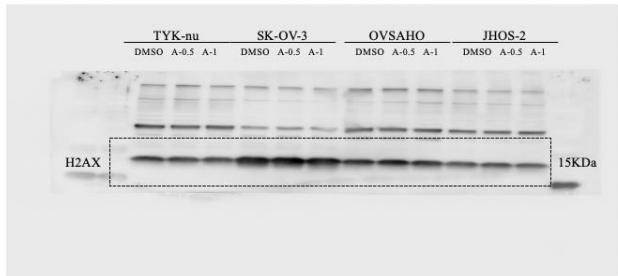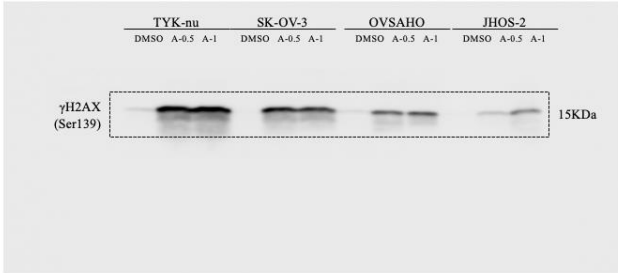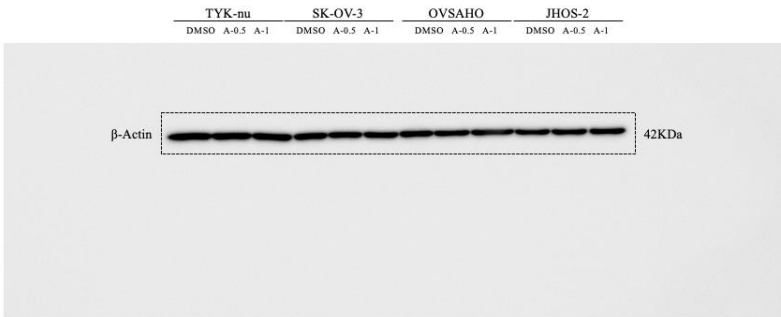

Fig. 4H

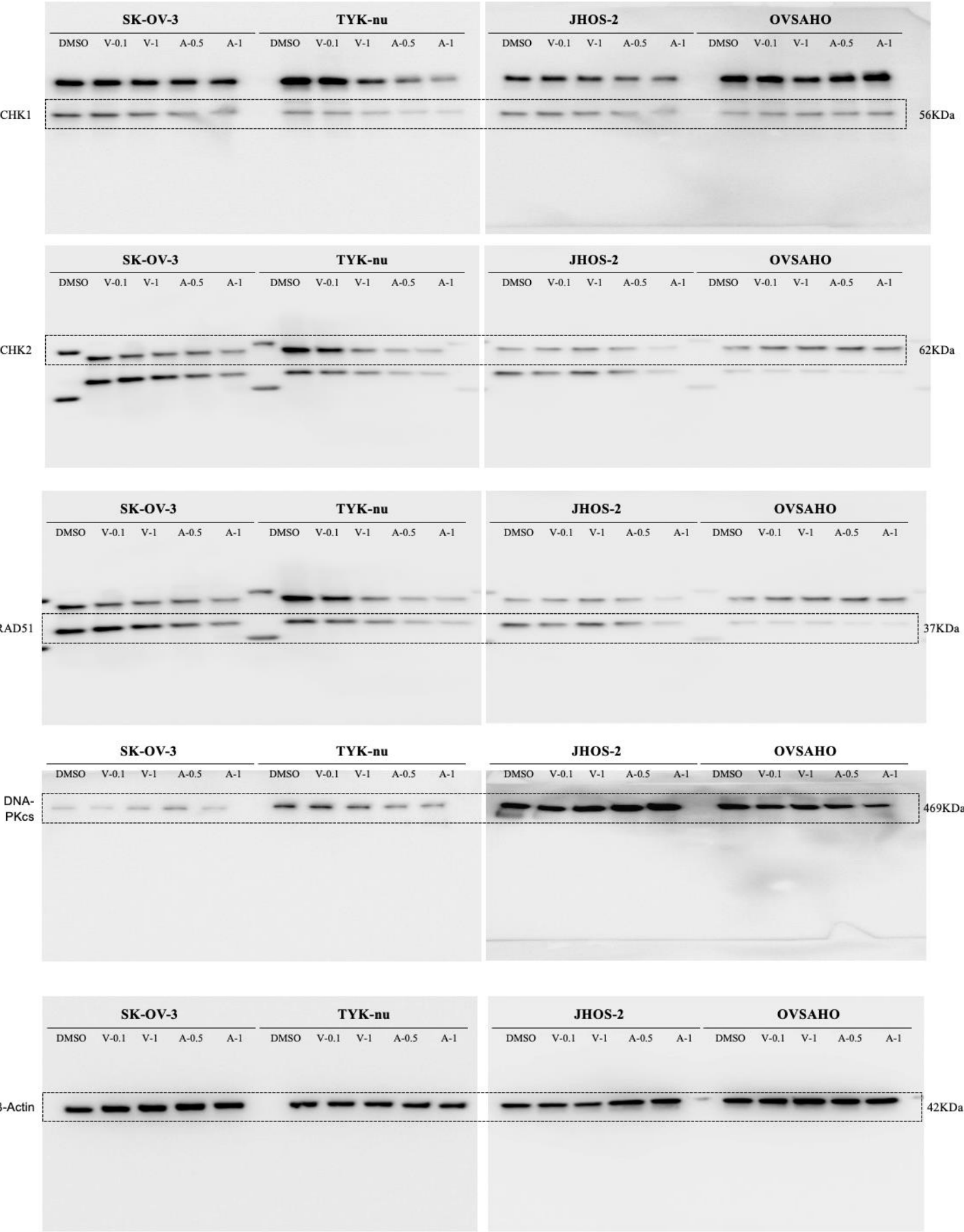

Fig. 5E

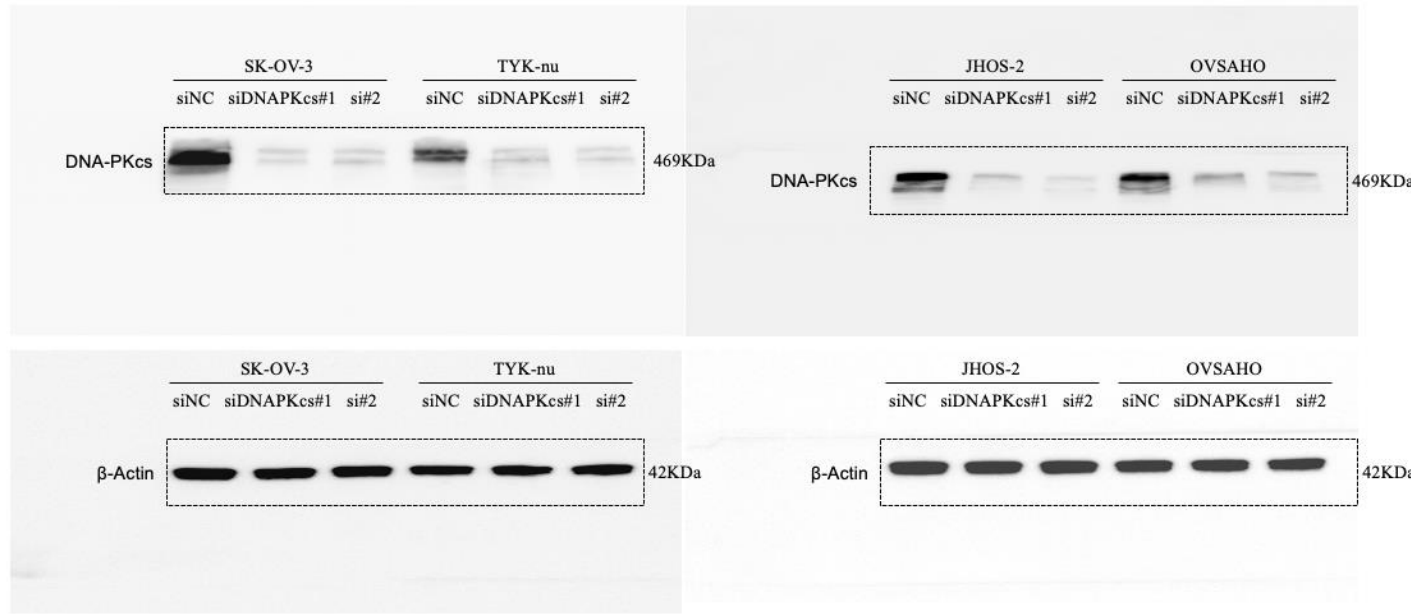

Fig. 6C

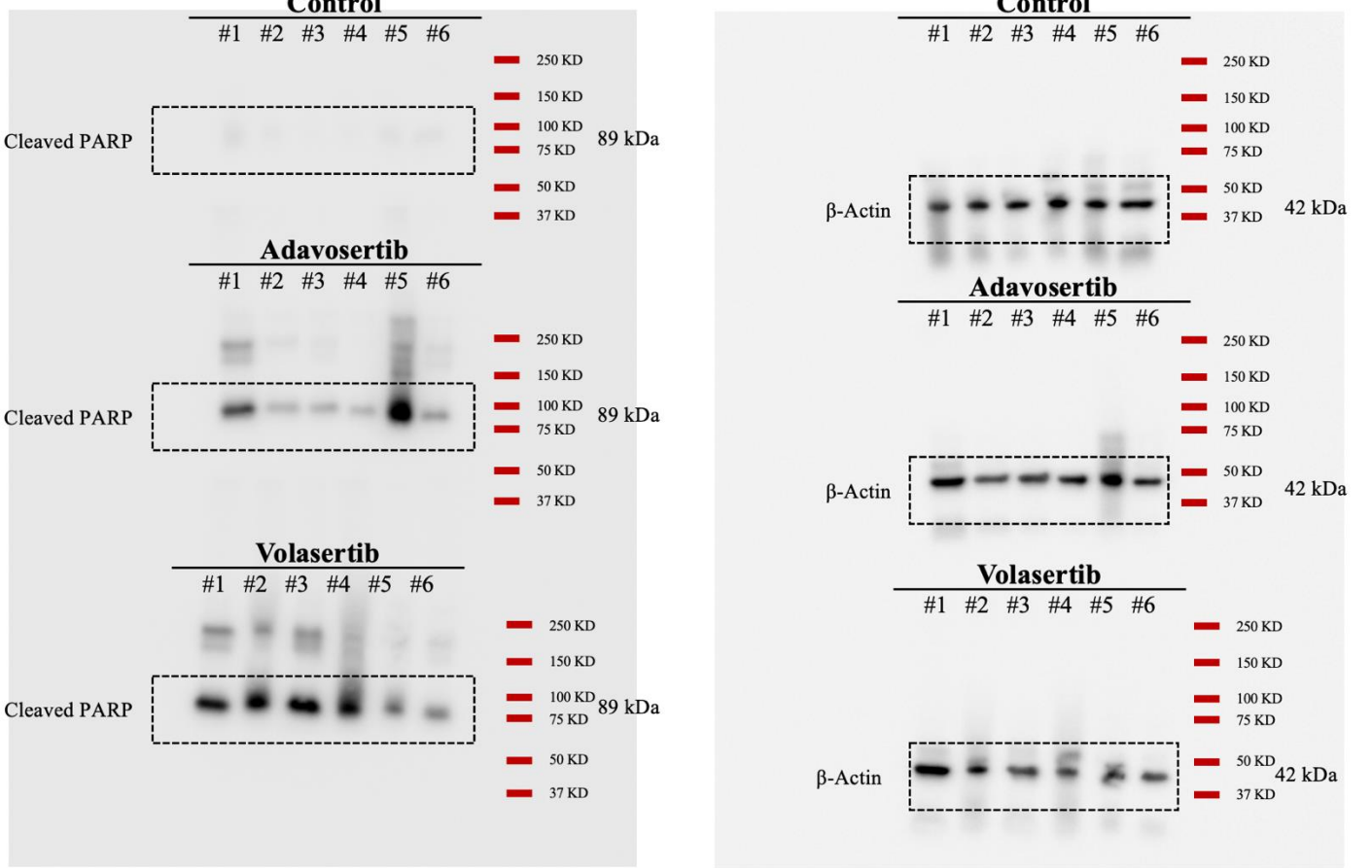

Fig. 6F

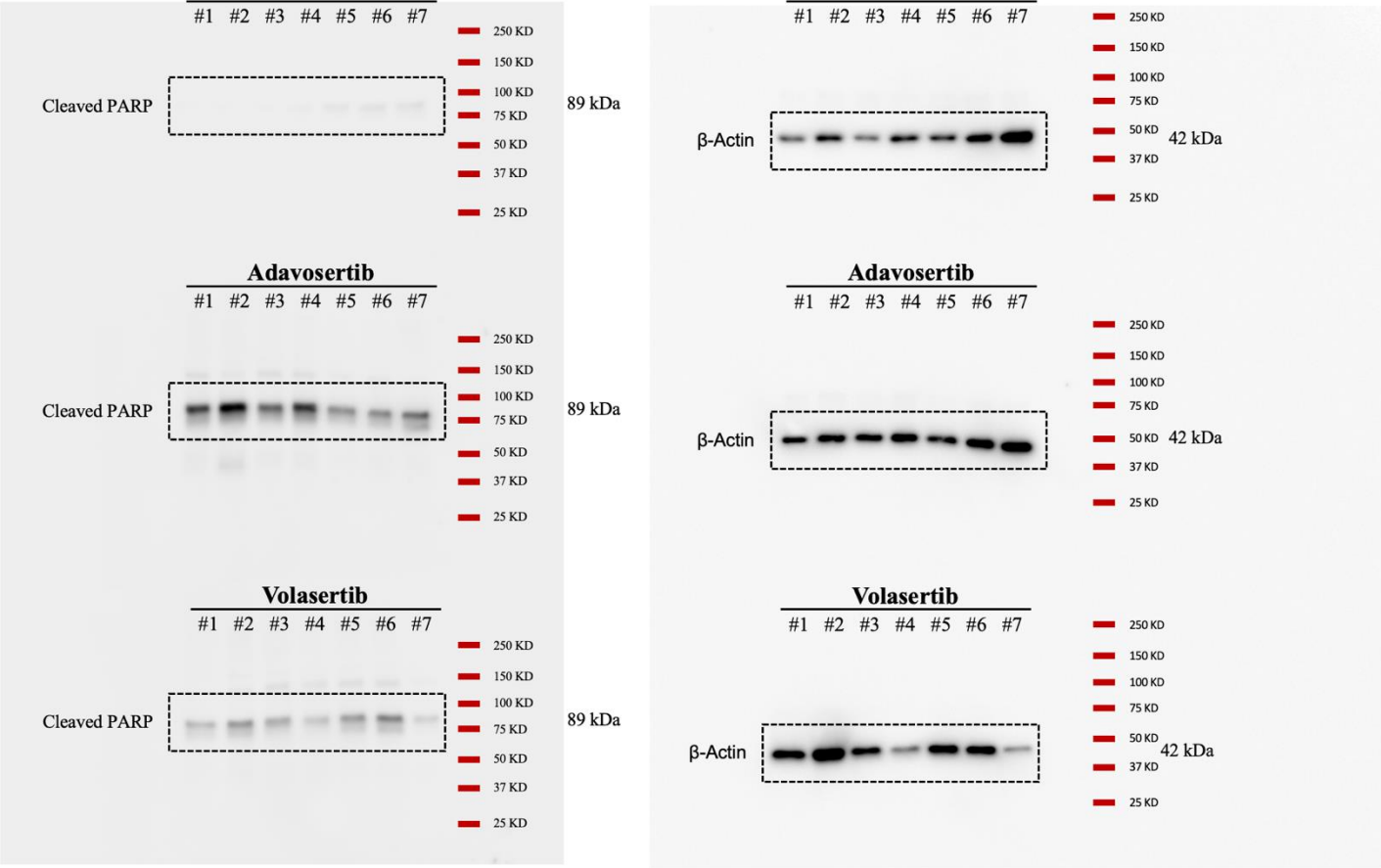

Fig. 6I

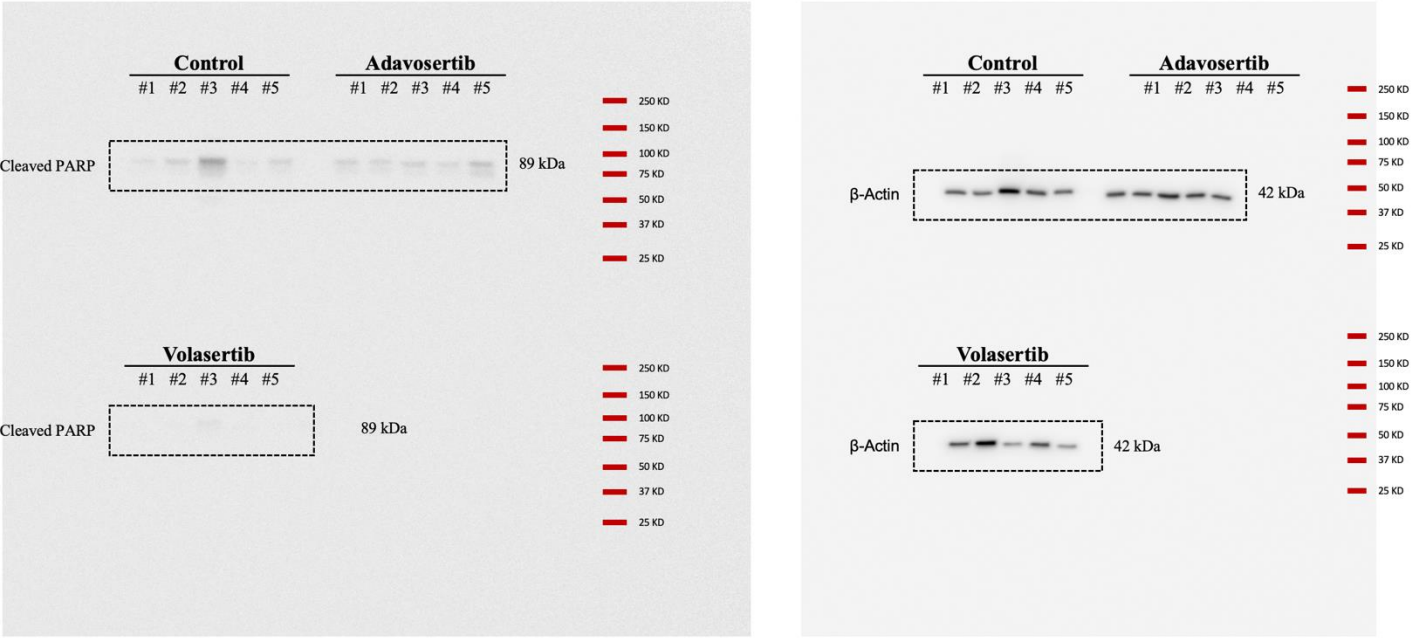

Supplement: Supplementary file 2 — Uncropped western blots [file 41419_2025_8324_MOESM2_ESM.pdf]
